# Spatial Origin and Diversification of the *Lycocerus fainanus* Species Group (Coleoptera, Cantharidae), with Descriptions of Four New Species from China and Vietnam †

**DOI:** 10.3390/insects12050445

**Published:** 2021-05-13

**Authors:** Huacong Xi, Younan Wang, Tong Liu, Xingke Yang, Haoyu Liu, Yuxia Yang

**Affiliations:** 1 The Key Laboratory of Zoological Systematics and Application, School of Life Science, Institute of Life Science and Green Development, Hebei University, Baoding 071002, China; xihuacong5253@163.com (H.X.); wangyounan@stumail.hbu.edu.cn (Y.W.); liutong@stumail.hbu.edu.cn (T.L.); 2 Key Laboratory of Zoological Systematics and Evolution, Institute of Zoology, Chinese Academy of Sciences, Beijing 100101, China; yangxk@ioz.ac.cn

**Keywords:** taxonomy, distribution, biogeography, *Lycocerus*, oriental region

## Abstract

**Simple Summary:**

*Lycocerus* Gorham, 1889 has about 320 species worldwide at present, of which a small part have been attributed into 13 species groups, but the rest remain uncertain in their status. In the present study, five previously known species and another four new species from China and Vietnam were classified to the *Lycocerus* *fainanus* species group, based on their morphological characters. Then the distribution pattern of this group was analyzed with ArcGIS techniques. It showed that all species were constricted to the subtropical area of the Oriental region and distributed in both Taiwan and the continent. Further, the ancestral distribution range was reconstructed with the aid of the Reconstruct Ancestral State in Phylogenies (RASP) software, based on a phylogeny of morphological data. The results showed that the spatial origin was located in northern Vietnam and southwest China. Subsequently, two dispersal routes occurred: the first originated from the continent and then to Taiwan, the second from Taiwan to the continent, and the divergence between the species was caused by dispersal and vicariance. Unfortunately, it was difficult to estimate the timing of origin because of the lack of direct fossil evidence and molecular data.

**Abstract:**

Five previously known species were attributed to the *Lycocerus fainanus* species group, including *L. inopaciceps* (Pic 1926), *L. oberthueri* (Gorham 1889), *L. oudai* (Švihla 2004), *L. metallipennis* (Fairmaire 1887), and *L. nigripes* (Wittmer, 1995). Four new species of this group were discovered from China and Vietnam, *L. binotatus* sp. nov., *L. testacicollis* sp. nov., *L. daliensis* sp. nov., and *L. vietnamensis* sp. nov. An updated key to all species was provided. A geographical distribution map is presented, which shows that all the members were located between 18.69041–33.93441° N, and between 98.61413–121.77102° E. The ancestral geographical range was reconstructed based on a phylogeny of morphological data by the Bayesian Binary MCMC method. The result showed that the spatial origin of *L. fainanus* species group was probably located in northern Vietnam and southwest China. The divergence of the species in southwest China and Taiwan was caused by vicariance about 24 Ma ago, when the latter was separated in the Qinghai-Tibet Plateau, and the remaining species of mainland China all originated from Taiwan after traveling around Southeast Asia and back to China. Nevertheless, this conclusion should be verified when fossil evidence and molecular data are available.

## 1. Introduction

The cantharid genus *Lycocerus* Gorham, 1889 belongs to the tribe Cantharini and the subfamily Cantharinae [1]. It was defined in a broad sense by Okushima [2], who regarded *Athemus* Lewis, 1895 and its subgenera, *Athemellus*, Wittmer 1972, *Andrathemus* Wittmer, 1978, *Mikadocantharis* Wittmer *et* Magis, 1978 and *Isathemus* Wittmer, 1995, as junior synonyms of *Lycocerus*. These taxa were originally discriminated from one another by different characteristics of tarsal claws or antennal configuration; however, this was suggested not to be definite or sufficient to determine genera or subgenera [2]. From then, the genus became one of the largest genera in Cantharidae, with about 320 species widely distributed in the Oriental and eastern Palaearctic Regions [1,3].

In the monograph of taxonomic study on *Lycocerus* from Japan by Okushima [2], nine species groups were recognized mainly based on the characteristics of genitalia in both sexes and phylogenies of the species groups and subgroups were analyzed. After that, another four species groups were proposed [4,5,6,7]. Among them, the *Lycocerus* *fainanus* species group was originally established as a subgroup of *L. vitellinus* species group [8], which is composed of four subgroups including eight species (subspecies) distributed in Japan and China, and was recently upgraded into an independent group [7]. The members of this group are characterized by the middle or large-sized body and metallic green or blue elytra, particularly the median lobe of aedeagus present with a conspicuous process directed dorsally at apex, as well as relatively long diverticulum and spermatheca but short spermathecal duct [7]. At present, nine species (subspecies) are included in this group, and most of them are endemic to Taiwan, except one subspecies, which is common on the Chinese mainland, and one species is spread through northern Vietnam.

During our study on *Lycocerus*, some species were found to belong *L. fainanus* species group; meanwhile, some new species were discovered. With more species added into this group, its distribution range extends much wider, with several provinces of southern China included, so its distribution pattern seems more interesting. 

Most species of *Lycocerus* are distributed in a narrow area, such as the Japanese members being mostly endemic [2], and those from Taiwan are restricted to the island [7,8,9]. They have limited flight capacity for a long-distance dispersal though the adults can fly; therefore, their speciation, as well as population structure, have a relatively strong dependence on geographical isolation [10,11,12,13,14,15]. Thus, standing as a model insect group to investigate the correlation between the distribution pattern and major geological events, the historical biogeography of *L. fainanus* species group is of considerable interest. In the present study, the geographical origin, specification, and migration for *L. fainanus* species group were supposed, by using Reconstruct Ancestral State in Phylogenies [16], based on a reconstructed phylogeny of morphological data.

## 2. Materials and Methods

### 2.1. Specimens Studied

Specimens examined are deposited in the following collections: Institute of Zoology, Chinese Academy of Sciences, Beijing, China (IZAS), Museum of Hebei University, Baoding, China (MHBU), the Institute of Entomology, College of Life Sciences, Nankai University, Tianjin, China (NKUM) and Zoological Institute, Russian Academy of Sciences, St. Petersburg, Russia (ZIN).

### 2.2. Terminology and Techniques

Morphological terminology used in this study mainly follows that of Okushima [2]. The abbreviations in the figures are as follows, female genitalia (ag: accessory gland; di: diverticulum; sd: spermathecal duct; sp: spermatheca;ov: median oviduct; va: vagina) and male genitalia (bp: basal piece; dp: dorsal plate of each paramere; is: inner sac of median lobe; lp: laterophyse; ml: median lobe; pm: process of median lobe; vp: ventral process of each paramere). 

The definition of *Lycocerus* *fainanus* species group follows Okushima and Hsiao [7]. The material was determined on the basis of the examination of type specimens, also with reference to the publication by Wittmer [17], as well as our own taxon concepts.

Genitalia of both sexes and abdominal sternites VIII of females were dissected and cleared in 10% NaOH solution, and female genitalia was dyed with hematoxylin. Habitus photos were taken by a Leica M205A stereomicroscope; multiple layers were stacked using Combine ZM (Helicon Focus 5.3). Line drawings were made using a camera lucida attached to a Nikon SMZ1500 stereomicroscope, then edited in CorelDRAW 12 and Adobe Photoshop CS3.10.0.1.

### 2.3. Taxon Sampling and Morphological Characters

All 18 species (including 4 new species) of *L. fainanus* species group were chosen as ingroups, and 2 species, *L. canthariformis* (Ishida 1986) and *L. pluricostatus* (Fairmaire 1887) of *L. vitellinus* species group as outgroups, since they were closely related because of the common characters in genitalia of both sexes [2]. In total, 34 morphological characters (Table S1) were included in the morphological data matrix (Table S2). Missing data were coded as ‘?’, and inapplicable data as ‘-’. All characters were treated as unordered and of equal weight.

### 2.4. Phylogenetic Analysis

The data matrix was analyzed using PAUP 4.0b [18] using the Neighbor-Joining method (using default algorithms). A separate maximum likelihood (ML) ultrafast bootstrap analysis was also performed in IQTREE [19] using the best-fit model MK + FQ + ASC + G4 for categorical data, and empirical character state frequencies with the correction for ascertainment bias applied.

Occurrence records of the species of the *L. fainanus* species group were obtained from relevant literature records [7,8,17] and the examined specimens in the present study. In total, 128 distribution records were collected (Table S3), and the distribution map was prepared with the ArcGIS 10.2 (ESRI Inc., California, LA, USA) and processed in the Adobe Photoshop CS5.

According to the distribution information of the species, combined with the biogeographical regions [20], the geographical divisions of the *L. fainanus* speciesgroup were recognized. Based on the phylogenetic tree and the geographical divisions, the ancestral biogeographic areas were reconstructed on the most parsimonious trees from analysis of equally-weighted characters using the Bayesian Binary MCMC (BBM) method in RASP 4.2 [16] under the default settings. Taxa that occured in more than one region were assigned multiple states according to known distributions.

## 3. Results

### 3.1. Taxonomy

The Lycocerus fainanus species group

**Figure 1:**
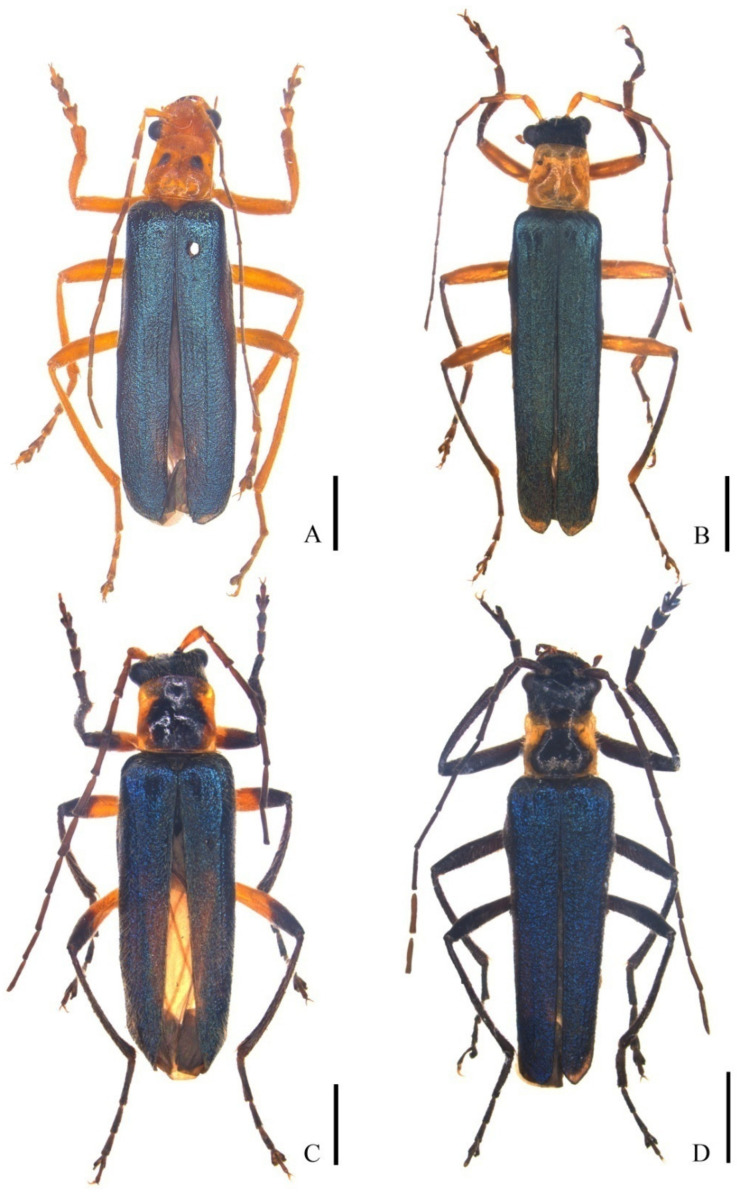
Male habitus, dorsal view: (**A**) *Lycocerus* *bimaculaticollis* **sp. nov.**; (**B**) *L. testacicollis* **sp. nov.**; (**C**) *L. daliensis* **sp. nov.**; (**D**) *L. vietnamensis* **sp. nov.** Scale bars: (**A**) 2.0 mm; (**B**–**D**) 5.0 mm.

**Figure 2:**
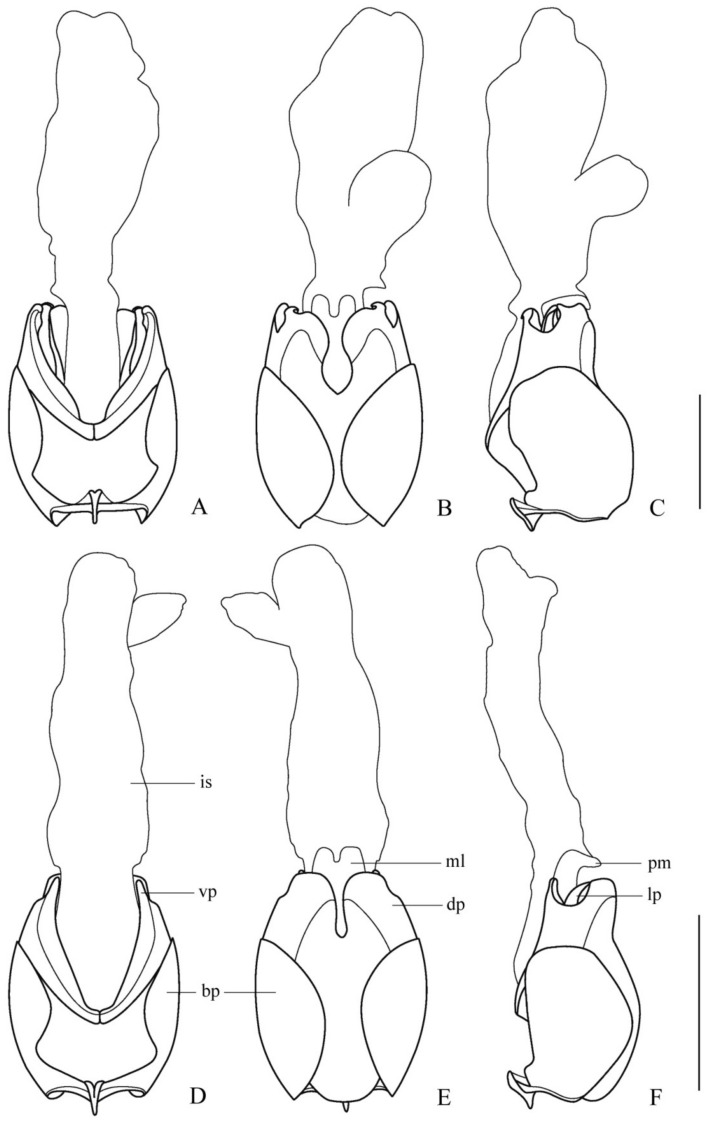
Aedeagus (**A**,**D**). ventral view; (**B**,**E**) dorsal view; (**C**,**F**) lateral view): (**A**–**C**) *Lycocerus* *binotatus* **sp. nov.**; (**D**–**F**) *L. testacicollis* **sp. nov.** Scale bars: 1.0 mm.

**Figure 3:**
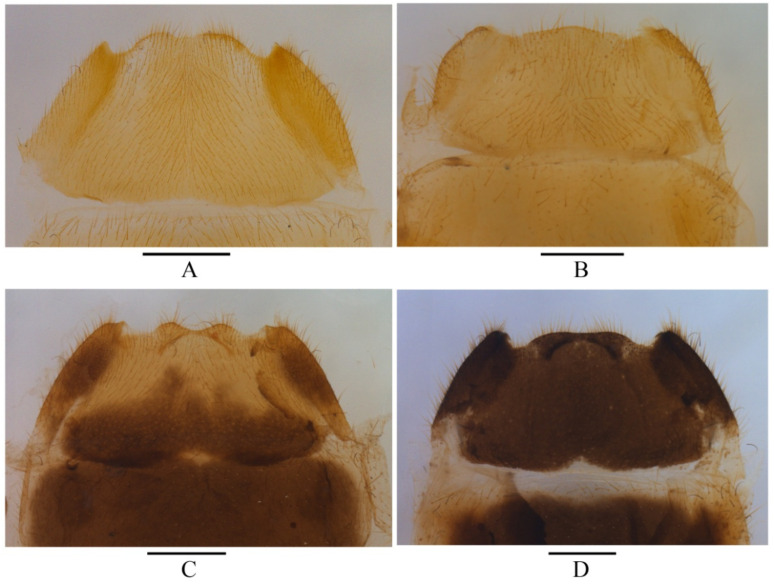
Abdominal sternite VIII of female, ventral view: (**A**) *Lycocerus* *binotatus* **sp. nov.**; (**B**) *L. testacicollis* **sp. nov.**; (**C**) *L. daliensis* **sp. nov.**; (**D**) *L.* *vietnamensis* **sp. nov.** Scale bars: 0.5 mm.

**Figure 4:**
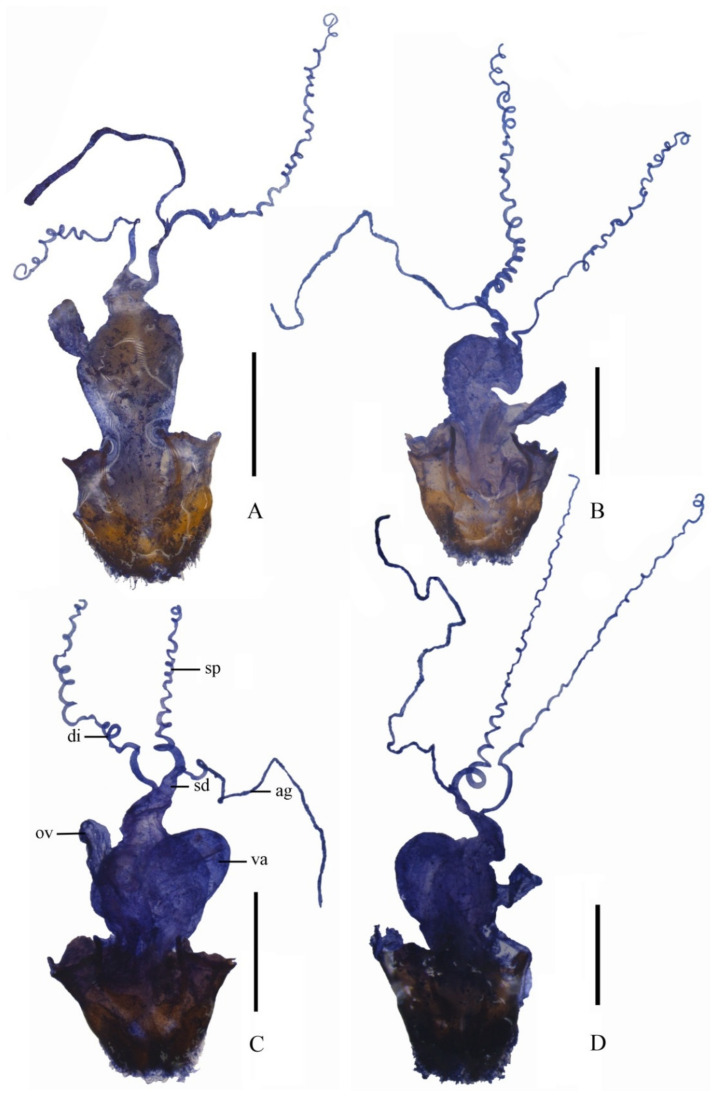
Female internal genitalia, lateral view: (**A**) *Lycocerus* *binotatus* **sp. nov.**; (**B**) *L. testacicollis* **sp. nov.**; (**C**) *L. daliensis* **sp. nov.**; (**D**) *L.* *vietnamensis* **sp. nov.**; Scale bars: 1. 0 mm.

**Figure 5:**
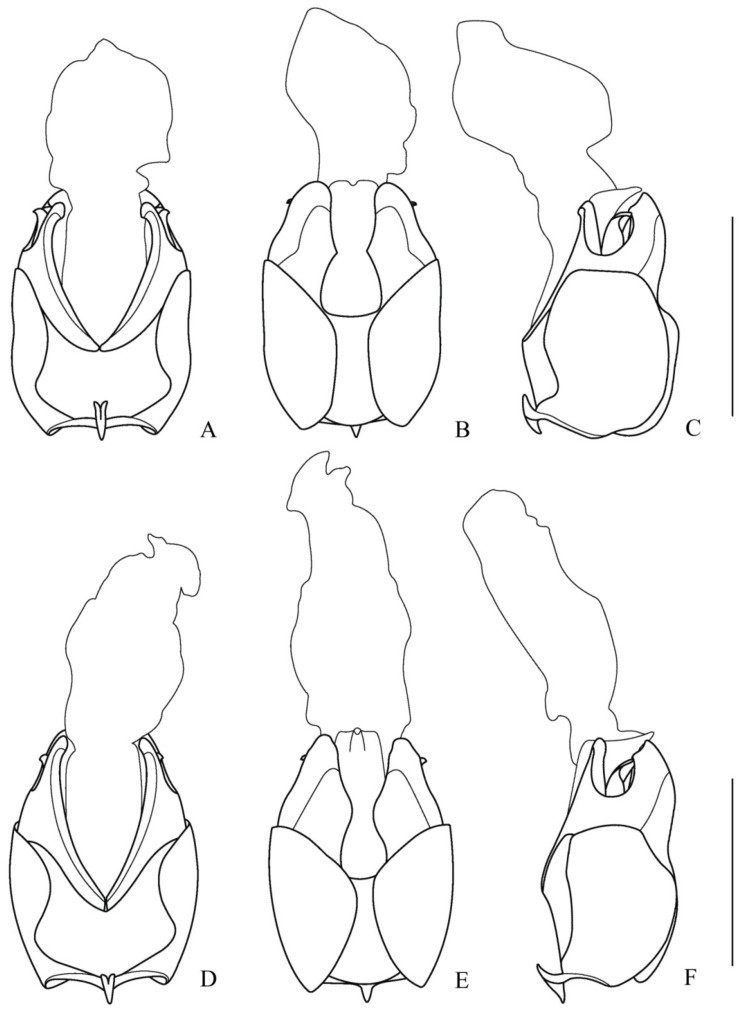
Aedeagus (**A**,**D**) ventral view; (**B**,**E**) dorsal view; (**C**,**F**) lateral view): (**A**–**C**) *Lycocerus* *daliensis* **sp. nov.**; (**D**–**F**) *L. vietnamensis* **sp. nov.** Scale bars: 1.0 mm.

**Remarks**. According to the definition of Okuhisma and Hsiao [7], five species from the Chinese mainland are attributed to the *L. fainanus* species group, including *L. inopaciceps* (Pic, 1926), *L. oberthueri* (Gorham, 1889), *L. oudai* (Švihla, 2004), *L. metallicipennis* (Fairmaire, 1887), and *L. nigripes* (Wittmer, 1995). The above species, *L. metallescens fukienensis* (Wittmer, 1954), *L. fainanus* (Pic, 1910) and *L. taoyuanus* (Wittmer, 1983), are illustrated with aedeagi to show the characteristics of the median lobe (Supplementary Figures S1–S4) and abdominal sternites VIII of female (Supplementary Figure S5), and some were present with the female internal genitalia for the first time (Supplementary Figure S6). Besides, *L. satoi* Okushima, 2007 and *L. rufomandibularis* (Pic, 1914) are provided with some additional distribution information. Additionally, another four new species were discovered and described under the names of *L. binotatus* sp. nov. (Hainan, China), *L. testacicollis* sp. nov. (Guangxi, China), *L. daliensis* sp. nov. (Yunnan, China), and *L. vietnamensis* sp. nov. (Sa Pa, Vietnam) (Figure 1, Figure 2, Figure 3, Figure 4 and Figure 5). 

Now, 18 species (subspecies) are included in the *L. fainanus* species group, and they could be distinguished by the following key.

Key to the species in the *L. fainanus* species group (Figure 1, Figure 2, Figure 3, Figure 4 and Figure 5 and S1–S6).
1.All tarsal claws simple in female (Okushima and Hsiao [7]: Figure 2G,H)2- Pro- and meso-outer claws each with a digitiform tooth in female (Okushima and Hsiao [7]: Figure 2E,F)52.Body stout, pronotum subquadrate in male; abdominal sternite VIII of female triangularly emarginate at lateroapical angles (Supplementary Figure S5C):*L. inopaciceps* (Pic, 1926)- Body slender, pronotum longer than wide in male; abdominal sternite VIII of female rounded at lateroapical angles33.Head and legs uniformly black; aedeagus: laterophyse shorter than the ventral process of each paramere (Okushima and Hsiao [7]: Figure 4B)*L. niisatoi* Okushima *et* Hsiao, 2017;- Head at least orange at clypeus, legs uniformly orange or mixed orange with black; aedeagus: laterophyse nearly as long as the ventral process of each paramere (Supplementary Figure S2F)44.Legs uniformly orange*L. metallescensmetallescens* (Gorham, 1889)- Legs mixed orange with black at femora, sometimes also at tibiae and tarsi*L. metallescensfukienensis* (Wittmer, 1954).5.Antennae absent with grooves in middle antennomeres in males (Okushima and Hsiao [7]: Figure 2A)6- Antennae present with grooves in middle antennomeres in males (Okushima and Hsiao [7]: Figure 2C)76.Pronotum yellow on both sides of the disc (Figure 1D); aedeagus: the ventral process of each paramere is nearly as long as the dorsal plate (Figure 5F), which is moderately narrowed apically and roundly protuberant in the middle of the inner margin (Figure 5E); abdominal sternite VIII of female (Figure 3D) nearly straight in the middle of posterior margin*L. vietnamensis* sp. nov- Pronotum dark brown on both sides of the disc (Okushima and Hsiao [7]: Figure 1A,B); aedeagus: the ventral process of each paramereis longer than ventral process (Okushima and Hsiao [7]: Figure 3B), which is strongly narrowed apically and nearly straight at the inner margin (Okushima and Hsiao [7]: Figure 3C); abdominal sternite VIII of female (Okushima and Hsiao [7]: Figure 3D) roundly emarginate in middle of posterior margin*L. rufomandibularis* (Pic, 1914)7.Aedeagus: the ventral process of each paramere is acutely hooked at apices (Supplementary Figure S1D; Okushima [8]: Figures 4, 9 and 12)8- Aedeagus: the ventral process of each paramere is rounded at apices118.Head uniformly black; aedeagus: the dorsal plate of each paramere shorter than the ventral process, laterophyse reduced, not exceeding over the bottom of the emargination between the ventral process and dorsal plate, which is lower than the apical margin of the basal piece (Supplementary Figure S1F)*L. taoyuanus* (Wittmer, 1983)- The head is mixed with black and orange; aedeagus: the dorsal plate of each paramere is longer than the ventral process; laterophyse moderately or well-developed, exceeding over the bottom of the emargination between the ventral process and the dorsal plate, which is far from the apical margin of the basal piece (Okushima and Hsiao [7]: Figures 5, 10 and 13) 99.Elytra light yellow at lateral margins (Okushima [8]: Figure 2)*L. flavimarginalis* Okushima, 2007- Elytra uniformly metallic green1010.Vertex orange, tibiae black, pronotum darkened at the anterior part of the disc (Okushima [8]: Figure 3)*L. satoi,* Okushima, 2007- Vertex black, tibiae mixed with black and orange, pronotum darkened in middle of the disc (Okushima [8]: Figure 1)*L. masatakai* Okushima, 2007.11.Pronotum uniformly yellow or orange, or with a pair of small black markings on the disc12- Pronotum yellow, with a black marking in the center of the disc which always extends to the margins1412.Body slender; aedeagus: the ventral process of each paramere is nearly straight at the apex from a ventral view (Figure 2D); abdominal sternite VIII of female not emarginate in the middle of posterior margin (Figure 3B)*L. testacicollis* sp. nov.- Body massive; aedeagus: the ventral process of each paramere is obviously bent inwards at the apex in a ventral view (Figures 1A and S1A); abdominal sternite VIII of the female is roundly emarginate in the middle of the posterior margin (Figures 3A and S5A)1313.Head and legs uniformly orange; aedeagus: the ventral process of each paramere dorsal plate is gradually narrowed apically (Figure 2A); the dorsal plate is protuberant at a lateroapical angle in a dorsal view (Figure 2B)*L. binotatus* sp. nov.- Head and legs uniformly black or sometimes mixed with orange; aedeagus: the ventral process of each paramere is abruptly narrowed at the apical part (Supplementary Figure S1A); the dorsal plate is not protuberant at a lateroapical angle in a dorsal view (Supplementary Figure S1B)*L. fainanus* (Pic, 1910)14.Aedeagus: the dorsal plate of each paramere is separated far from each other in dorsal view (Figure 5B); abdominal sternite VIII of the female and the middle emargination is as wide as the lateral ones (Figure 3C)*L. daliensis* sp. nov- Aedeagus: the dorsal plate of each paramere approaches the each other in a dorsal view (Supplementary Figures S3B,E and S4B,E); abdominal sternite VIII of the female with the middle emargination much narrower than the lateral ones (Supplementary Figure S5E–H)1515.Aedeagus: the dorsal plate of each paramereis nearly as long as the ventral process, with a protuberance at the outer margin in a lateral view (Supplementary Figure S2F) *L. oudai* (Švihla, 2004)- Aedeagus: the dorsal plate of each paramere longer than the ventral process, with two protuberances at the outer margin in a lateral view (Supplementary Figures S3C and S4C,F)1616.Pronotum with a small black marking restricted to the center of the disc; legs orange, black at the apices of the femora and tarsi*L. oberthueri* (Gorham, 1889)- Pronotum with a large black marking, almost extending to the margins; legs uniformly black1717.Aedeagus: the dorsal plate of each paramere is obviously longer than ventral process (Supplementary Figure S6C); abdominal sternite VIII the of female (Supplementary Figure S5G) is not membranous at lateral emargination, with the portion between middle and lateral emarginations acute at apex*L. metallipennis* (Fairmaire, 1887)- Aedeagus: the dorsal plate of each paramere hardly longer than ventral process (Supplementary Figure S4F); abdominal sternite VIII of female (Supplementary Figure S5H) membranous at the lateral emarginations, with the portion between middle and lateral emarginations truncated at the apex*L. nigripes* (Wittmer, 1995)


#### 3.1.1. *Lycocerus* *binotatus* Y. Yang et X. Yang, sp. nov.

Figure 1A, Figure 2A–C, Figure 3A and Figure 4A

**Description.** Body length: 15.0–16.0 mm (15.0 mm in holotype); width: 3.0–4.5 mm (3.5 mm in holotype).

Male (Figure 1A). Body orange, apices of mandibles dark brown, antennae brown, except antennomeres I–II orange, pronotum with a pair of small black markings on disc, elytra green, with a strong metallic luster. Body densely covered with yellow recumbent pubescence, which is slightly sparser on head and pronotum than other parts, and slightly longer on clypeus than other parts.

**Head.** Evenly narrowed behind eyes, surface densely and finely punctate; eyes moderately protruding, head width across eyes slightly wider than the anterior margin of the pronotum; terminal maxillary palpomere nearly triangular, widest in the middle; antennae extending to the apical quarter length of elytra when reclined, antennomeres II shortest, about twice longer than wide at apex, III–IX slightly widened apically, V longest, X–XI nearly parallel-sided, XI slightly longer than X and acute at apex, III–XI each with a narrow, smooth longitudinal groove along the middle part of the outer margin, which is longer on IV–VIII than that on III, or IX–XI.

**Pronotum.** Subquadrate, nearly as long as wide, widest near the base, anterior margin slightly arcuate, lateral margins nearly parallel and slightly sinuate, posterior margin nearly straight, anterior angles rounded, posterior angles nearly rectangular, disc convex on posterolateral parts, surface finely and slightly sparse punctate than that on the head.

**Elytra.** About 4.9 times longer than pronotum, 3.3 times longer than the width across the humeri, humeral width distinctly wider than the posterior margin of pronotum, outer margins nearly parallel, disc mat, coarsely and densely punctuate, present with two slightly distinct longitudinal costae near the inner margins. Legs slender, femora and tibiae nearly straight, all tarsal claws simple.

**Aedeagus** (Figure 2A–C). Ventral process of each paramere slightly bent inwards in ventral view, with apex obtusely hooked; dorsal plate slightly longer than ventral process, with inner angle, widely rounded and the inner margin sinuate in dorsal view, outer angle triangularly protuberant and ridged on the inner surface, which accommodating apex of laterophyse, the outer margin is arcuate in lateral view; the bottom of lateral emargination between ventral process and dorsal plate far from apical margin of the basal piece; median lobe provided with a conspicuous process directed dorsally at the apex, with inner sac lengthened and swollen apically, longer than tegmen; laterophyse slightly longer than ventral process, as long as dorsal plate, bent dorsally, with apex indistinctly hooked and slightly directing outwards.

**Female.** Similar to males, but body larger, eyes less protruding, antennae shorter and narrower, extending to elytral mid-length when reclined, pronotum slightly wider than long, disc slightly convex, pro-and meso-outer tarsal claws each with a digitiform tooth at the base. Abdominal sternite VIII (Figure 3A) roundly emarginate on both sides and in middle of posterior margin, with lateral emarginations deeper than the middle one, the portion between lateral and middle emarginations rounded at apex, lateroapical angle acute at apex.

**Internal organ ofthe reproductive system** (Figure 4A). Vagina stout and abruptly thinned at ventroapical portion into a stout tube, where diverticulum and spermathecal duct arising separately; diverticulum moderately long, thin and spiral; spermathecal duct thicker and shorter than diverticulum; spermatheca provided with a spiral tube, gradually thinned apically, much longer than diverticulum; basal portion of spermatheca extended into a very short sharply-ended tube, at the opening of the accessory gland; accessory gland thin and much shorter than spermatheca.

**Type material.** Holotype:♂, China, Hainan, Wuzhishan, Changjiang, 922 m, 18°54.177′ N, 109°41.286′ E, 6.iv.2016, Chang Lingxiao and Bai Xinglong (MHBU); Paratypes: 2♂♂, same data as holotype (MHBU); 2♀♀, Hainan, Jianfengling, Mingfeng Valley, 969 m, 18°44.627′ N, 108°50.620′ E, 10.iv.2016, Chang Lingxiao and Bai Xinglong (MHBU); 1♂, Hainan, Wuzhishan Nature Reserve, 708 m, 18.90° N, 109.67° E, 10.iv.2010, Zhang Kuiyan (IZAS); 1♀, Hainan, Wuzhishan Nature Reserve, 708 m, 18.90° N, 109.67° E, 10.iv.2010, Zhang Kuiyan (IZAS); 1♂, Hainan, Jianfeng, Tianchi, 900 m, 13.iv.1980, Wang Shuyong (IZAS); 1♂, Hainan, Jianfeng, Tianchi, 900 m, 11.iv.1980, Pu Fuji (IZAS); 1♂, Hainan, Jianfeng, Tianchi, 900 m, 11.iv.1980, Wang Shuyong (IZAS); 1♀, Hainan, Jianfeng, 750 m, 25.iii.1980, Wang Shuyong (IZAS); 1♀, Hainan, Jianfengling, 22.iii.1984, Song Shimei (IZAS); 1♂, Hainan, Jianfengling Nature Reserve, Tianchi, 800 m, 30.iii.2003, Cai Bo (NKUM); 1♀, Hainan, Jianfengling Nature Reserve, Tianchi, 800 m, 2.iv.2003, Zhu Guangping and Cai Bo (NKUM); 2♀♀, Hainan, Bawangling Nature Reserve, East No.2 Management Station, 9.iv.2008, Zhu Guangping (NKUM).

**Etymology.** The specific name is derived from the prefix *bi*- (two) and suffix *notatus* (marked), referring to its pronotum with two small black markings on the disc.

**Description.** This new species is similar to *L. fainanus* (Pic, 1910) (located in Taiwan), but can be distinguished by the uniformly orange head, pronotum with a pair of small black markings on the disc, while the head is uniformly black or at least black on the vertex, pronotum black along the anterior margin or with a black marking on the center of the disc in the latter; aedeagus: distance between outer margins of dorsal plates narrower than that of ventral processes, while converse in the latter (Figures S1A‒C); abdominal sternite VIII of the female with shallow middle emargination, lateroapical angles are acute at apices, while middle emargination is moderately deep, lateroapical angles truncate at apices in the latter (Supplementary Figure S5A); diverticulum, spermatheca, and the accessory gland are shorter than those of the latter (Supplementary Figure S3A).

**Distribution:** China (Hainan).

#### 3.1.2. *Lycocerus* *testacicollis* Y. Yang et H. Liu, sp. nov.

Figure 1B, Figure 2D–F, Figure 3B and Figure 4B

**Description.** Body length: 11.0–12.0 mm (11.5 mm in holotype); width: 2.0–2.5 mm (2.3 mm in holotype).

**Male** (Figure 1B). Body yellow, mandibles dark brown, antennae black-brown, but antennomeres I–II yellow; elytra green, with a strong metallic shine; tibiae and tarsi black brown. Body densely covered with yellow recumbent pubescence, which is slightly sparser on head and pronotum than other parts, and slightly longer on clypeus than other parts.

**Head.** Evenly narrowed behind eyes, surface densely and finely punctate; eyes moderately protruding, head width across eyes slightly wider than the anterior margin of the pronotum; terminal maxillary palpomere nearly triangular, widest in the middle; antennae extending to apical one-half length of elytra when reclined, antennomeres II shortest, II–IX slightly widened apically, X–XI nearly parallel-sided, XI slightly longer than X and acute at apex, IV–XI each with a narrow, smooth longitudinal groove along the middle part of the inner margin, which is longer on IV–VIII than that on IX–XI.

**Pronotum.** Subquadrate, nearly as long as wide, anterior and posterior margins nearly straight, lateral margins nearly parallel and slightly sinuate, anterior angles slightly rounded, posterior angles nearly rectangular, disc moderately convex on posterolateral parts, surface finely and slightly sparse punctate than that on the head.

**Elytra**. About 5.0 times longer than pronotum, 3.8 times longer than the width across humeri, humeral width distinctly wider than the posterior margin of pronotum, outer margins nearly parallel, finely and densely punctuate, longitudinal costae hardly visible. Legs slender, femora nearly straight, tibiae weakly arcuate, all tarsal claws simple.

**Aedeagus** (Figure 2D–F). Ventral process of each paramere nearly straight in ventral view, with apex rounded; dorsal plate nearly as long as ventral process, with inner angles rounded and inner margins straight in dorsal view, outer angles obtuse-angled and outer margins slightly arcuate in lateral view; the bottom of lateral emargination between ventral process and dorsal plate far from apical margin of the basal piece; median lobe provided with a conspicuous process directed dorsally at apex, inner sac lengthened apically, much longer than tegmen; laterophyse nearly as long as the ventral process and dorsal plate, gradually narrowed apically and bent dorsally.

**Female.** Similar to males, but body slightly larger, eyes less protruding, antennae shorter, extending to elytral one-third length when reclined, disc indistinctly convex, pro- and meso-outer tarsal claws with a digitiform tooth at their base. Abdominal sternite VIII (Figure 3B) widely and roundly emarginate on both sides of posterior margin, lateroapical angles nearly rectangular.

**Internal organ of the reproductive system** (Figure 4B). Vagina stout, diverticulum and spermathecal duct arises separately at the ventroapical portion; diverticulum long, thin and spiral; spermathecal duct thicker and shorter than diverticulum; spermatheca provided with a spiral tube, gradually thinned apically, slightly longer than diverticulum; basal portion of spermatheca extended into a short round-ended tube, at the opening of the accessory gland; accessory gland thin and slightly shorter than spermatheca.

**Type material.** Holotype: ♂, China, Guangxi, Wuming, Damingshan, 1100 m, 27.v.2011, Liu Haoyu (MHBU); Paratypes:2♀♀, Guangxi, same data as holotype (MHBU); 2♂♂, 1♀, Guangxi, Wuming, Damingshan, 1230–1423 m, 20.v.2011, Liu Haoyu (MHBU).

**Etymology.** The specific name is derived from Latin *testaceus* (of a brick-brownish- yellow color) and *collum* (neck), referring to its uniformly yellow pronotum.

**Diagnosis.** This new species is similar to *L. oberthueri* (Gorham, 1889) but can be distinguished by the no marking pronotum, while pronotum with a black marking on the center of the disc in the latter; aedeagus: ventral process distinctly shorter than that of the latter, dorsal plate nearly as long as ventral process, while dorsal plate distinctly longer than the ventral process in the latter (Supplementary Figures S6A‒C); abdominal sternite VIII of the female with no middle emargination, while distinctly triangularly emarginate in middle of posterior margin in the latter (Supplementary Figure S5E); basal portion of spermatheca extend into a short round-ended tube, at the opening of the accessory gland; while the accessory gland directly opens at the basal portion of the spermatheca in the latter (Supplementary Figure S6C).

**Distribution:** China (Guangxi).

#### 3.1.3. *Lycocerus* *daliensis* Y. Yang et X. Yang, sp. nov.

Figure 1C, Figure 3C, Figure 4C and Figure 5A–C

**Description.** Body length: 11.0–12.5 mm (11.0 mm in holotype); width: 2.8–3.4 mm (2.8 mm in holotype).

**Male** (Figure 1C). Body yellow, mandibles, maxillary palpi, and claws dark brown, antennae dark brown, antennomeres I and ventral side of II yellow, pronotum yellow, with large black markings extending from anterior margin to posterior margin, elytra green, with a strong metallic luster, legs black, coxae, trochanters and basal parts of femora yellow. Body densely covered with yellow recumbent pubescence, which is slightly sparser on head and pronotum than on other parts, and slightly longer on clypeus than other parts.

**Head.** Evenly narrowed behind eyes, surface densely and finely punctate; eyes moderately protruding, head width across eyes slightly wider than the anterior margin of pronotum; terminal maxillary palpomere nearly triangular, widest in the middle; antennae extending to the apical three-quarter length of the elytra when reclined, antennomeres II shortest, about twice longer than wide at apex, III–X slightly widened apically, XI nearly parallel-sided, XI slightly longer than X and acute at apex, IV–XI each with a short, narrow, smooth longitudinal groove along the middle part of the inner margins.

**Pronotum.** Subquadrate, nearly as long as wide, anterior margin slightly arcuate, lateral margins nearly parallel and slightly sinuate, posterior margin nearly straight, anterior angles rounded, posterior angles nearly rectangular, disc moderately convex on posterolateral parts, surface finely and slightly sparse punctate than that on the head.

**Elytra**. About 4.1 times longer than pronotum, 2.8 times longer than the width across humeri, humeral width distinctly wider than the posterior margin of pronotum, outer margins nearly parallel, disc coarsely and densely punctuate, longitudinal costae hardly visible. Legs slender, femora nearly straight, tibiae weakly arcuate, all tarsal claws simple.

**Aedeagus** (Figure 5A–C). Ventral process of each paramere bent inwards in ventral view, with apex obtusely hooked; dorsal plate longer than ventral process, with inner angle rounded and inner margin triangularly protuberant in the middle in dorsal view, outer angle nearly rectangular and outer margin arcuate in lateral view; the bottom of lateral emargination between ventral process and dorsal plate far from apical margin of the basal piece; median lobe provided with a conspicuous process directed dorsally at apex, inner sac lengthened and swollen apically, shorter than tegmen; laterophyse slightly shorter than ventral process, bent dorsally in lateral view, with apex distinctly hooked and directing outwards.

**Female**. Similar to the males, but body slightly larger, eyes less protruding, antennae shorter, extending to elytral mid-length when reclined, pronotum wider than long, pro-and meso-outer tarsal claws each with a digitiform tooth at the base. Abdominal sternite VIII (Figure 3C): roundly emarginate on both sides and in middle of posterior margin, middle emargination slightly shallower than the lateral ones, the portion between lateral and middle emarginations rounded at apex, lateroapical angle feebly emarginate at apex.

**Internal organ ofthe reproductive system** (Figure 4C). Vagina stout and abruptly thinned at the ventroapical portion, where diverticulum and spermathecal duct arising separately; diverticulum moderately long, thin and spiral; spermathecal duct distinctly shorter and thicker than diverticulum; spermatheca provided with a spiral tube, gradually thinned apically, nearly as long as diverticulum; basal portion of spermatheca extended into a short round-ended tube, at the opening of the accessory gland; accessory gland thin and slightly longer than the spermatheca.

**Type material.** Holotype: ♂, China, Yunnan, Dali, Cangshan, 30.v.1955, Yang Xingchi (IZAS); Paratypes: 3♀♀, same data as holotype (IZAS); 1♀, Yunnan, Dali, Zhonghefeng, 16.vi.1986, collector unknown (IZAS).

**Etymology.** The specific name is derived from the name of the type locality, Dali, Yunnan Province, China.

**Diagnosis.** This new species most resembles *L. vietnamensis* sp. nov. in the structure of aedeagus but can be easily separated from the latter by the presence of antennal grooves in males, bicolored antennae and femora, weakly arcuate tibiae, subquadrate pronotum, abdominal sternite VIII of female roundly emarginate in middle of posterior margin, with lateroapical angels weakly emarginate at apices. On the contrary, in *L. vietnamensis* sp. nov., antennal grooves in males are absent, antennae and femora are uniformly black, tibiae are nearly straight, the pronotum is longer than wide, and the abdominal sternite VIII of the female is nearly straight in the middle of posterior margin, whose lateroapical angles nearly truncate at apices.

**Distribution:** China (Yunnan).

#### 3.1.4. *Lycocerus* *vietnamensis* Y. Yang et H. Xi, sp. nov.

Figure 1D, Figure 3D, Figure 4D and Figure 5D–F

**Description.** Body length: 11.0–12.5 mm (11.0 mm in holotype); width: 2.8–3.4 mm (2.8 mm in holotype).

**Male** (Figure 1D). Body black, mandibles, and apices of maxillary palpi dark brown, pronotum yellow, black at anterior margin, with large black markings on posterior parts of the disc, elytra blue, with a strong metallic luster. Body densely covered with recumbent pubescence, which yellow on femora, clypeus, and margins of pronotum, black on other parts, the pubescence slightly sparser on head and pronotum than other parts, and slightly longer on clypeus than other parts.

**Head.** Slightly narrowed behind eyes, surface densely and finely punctate; eyes moderately protruding, head width across eyes nearly as long as anterior margin of pronotum; terminal maxillary palpomere nearly triangular, widest in the middle; antennae nearly extending to apices of elytra when reclined, antennomeres II shortest, about twice longer than wide at apex, III–VI slightly widened apically, VII–XI nearly parallel-sided, XI slightly longer than X and acute at apex.

**Pronotum.** Subquadrate, nearly as long as wide, anterior and posterior margins nearly straight, lateral margins nearly parallel and slightly sinuate, anterior angles slightly rounded, posterior angles nearly rectangular, disc moderately convex on posterolateral parts, surface finely and slightly densely punctate than that on the head.

**Elytra**. About 4.3 times longer than pronotum, 1.5 times longer than the width across humeri, humeral width distinctly wider than the posterior margin of pronotum, outer margins nearly parallel, disc coarsely and densely punctuate, present with two moderately developed longitudinal costae near the inner margins. Legs slender, femora and tibiae nearly straight, all tarsal claws simple.

**Aedeagus** (Figure 5D–F). Ventral process of each paramere slightly bent inwards in ventral view, with apex rounded; dorsal plate nearly as long as ventral process, inner angle rounded and inner margin roundly protuberant in the middle in dorsal view, outer angle nearly rectangular and outer margin nearly straight in lateral view; the bottom of lateral emargination between ventral process and dorsal plate near to apical margin of the basal piece; median lobe provided with a conspicuous process directed dorsally at the apex, inner sac lengthened apically, long than tegmen; laterophyse shorter than ventral process, bent dorsally in lateral view, with the apex distinctly hooked and directing outwards.

**Female**. Similar to males, but body larger, eyes less protruding, antennae shorter, extending to elytral mid-length when reclined, pronotum wider than long, disc slightly convex, pro- and meso-outer tarsal claws each with a digitiform tooth at their base. Abdominal sternite VIII (Figure 3D): roundly emarginate on both sides and nearly straight in the middle of posterior margin, lateroapical angle truncate at apex.

**Internal organ ofthe reproductive system** (Figure 4D). Vagina stout and abruptly thinned at the ventroapical portion, where diverticulum and spermathecal duct arising separately; diverticulum long, thin and spiral; spermathecal duct distinctly shorter and thicker than diverticulum; spermatheca provided with a spiral tube, gradually thinned apically, as long as diverticulum; basal portion of spermatheca extended into a short sharply-ended tube, at the opening of the accessory gland; accessory gland thin and nearly as long as spermatheca.

**Type material.** Holotype: ♂, Vietnam, Lao Cal Prov., Sa Pa Distr, Fan Si Pan Mt., 1900–2500 m, 22°20.58′ N, 103°46.15′ E, 20.iv-9.v.1999, Nikolai L. Orlov (ZIN); Paratypes: 3♀♀, 4♂♂ (2♂♂, 2♀♀ in ZIN; 2♂♂, 2♀♀ in MHBU), same data as holotype; 1♀, BьETHAM гоpыy ШA-ПA(Sa Pa, Vietnam), 1600–2000 m, 5.vi.1963, Kaбaков (Kabakov) (ZIN).

**Etymology.** The specific name is derived from the name of the type locality, Vietnam.

**Diagnosis.** This new species is most similar to *L. rufomandibularis* (Pic, 1914) in the absence of antennal grooves in males, but can be distinguished from the latter by the pronotum yellow on both sides of the disc (black brown in the latter; Okushima and Hsiao [7]: Figure 1A,B); aedeagus: the ventral process of each paramere nearly as long as the dorsal plate, which is moderately narrowed apically, roundly protuberant in middle of inner margin (in the latter species, the ventral process of each paramere longer than the dorsal plate, which strongly narrowed apically and nearly straight at inner margin; Okushima and Hsiao [7]: Figure 3B,C); the abdominal sternite VIII of female nearly straight in the middle of the posterior margin (roundly emarginate in the latter; Okushima and Hsiao [7]: Figure 3D).

Distribution: Vietnam.

### 3.2. Biogeography

#### 3.2.1. Distribution Patterns of *L. Fainanus* Species Group

Figure 6

**Figure 6:**
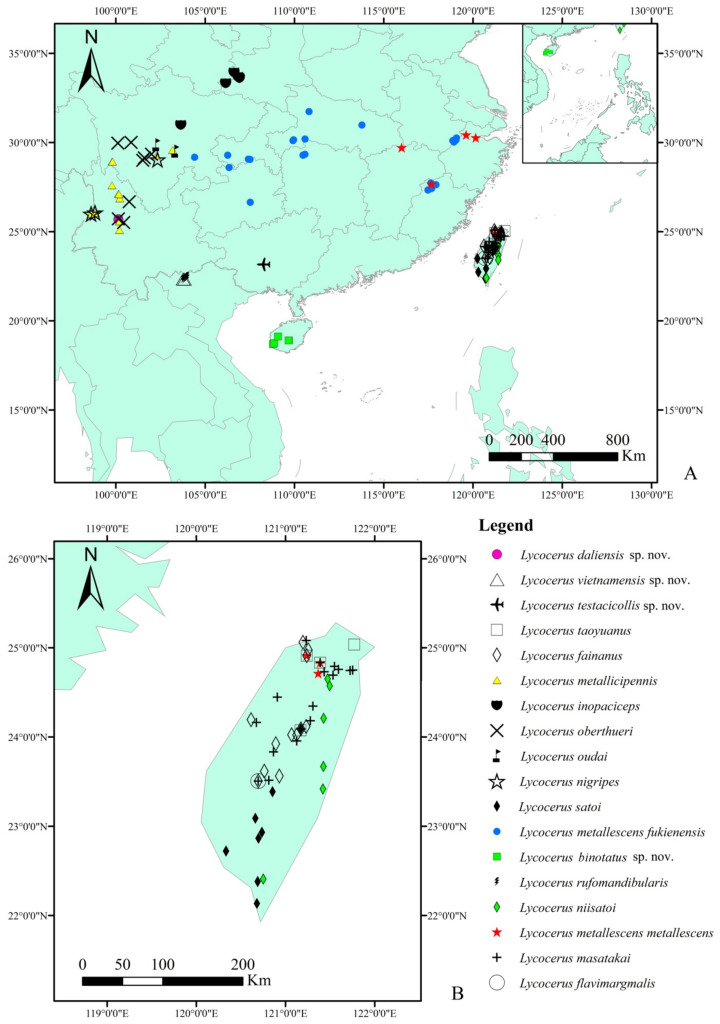
(**A**). Distribution map of *Lycocerus* *fainanus* species group in a general view; (**B**). Distribution map of the species from Taiwan in a close-up view.

Eight species are endemic to Taiwan (Figure 6B), and one to Hainan, two species constricted to northern Vietnam and one to Guangxi, China, *L. metallescens* *metallescens* is commonly found in Taiwan and southeastern China, while *L. metallescens* *fukienensis* is widely distributed in southeastern and central China, and the rest are concentrated in southwest China.

All of the species are distributed in the south of the Qinling Mountains, Huaihe River Line [21], located between 18.69041–33.93441° N and 98.61413–121.77102° E (Figure 6A), where the climate is subtropical. Thus, it is presumed that the members of *L. fainanus* species group prefer a warm and rainy climate.

#### 3.2.2. Ancestral Geographical Range Reconstruction

Figure 7

**Figure 7:**
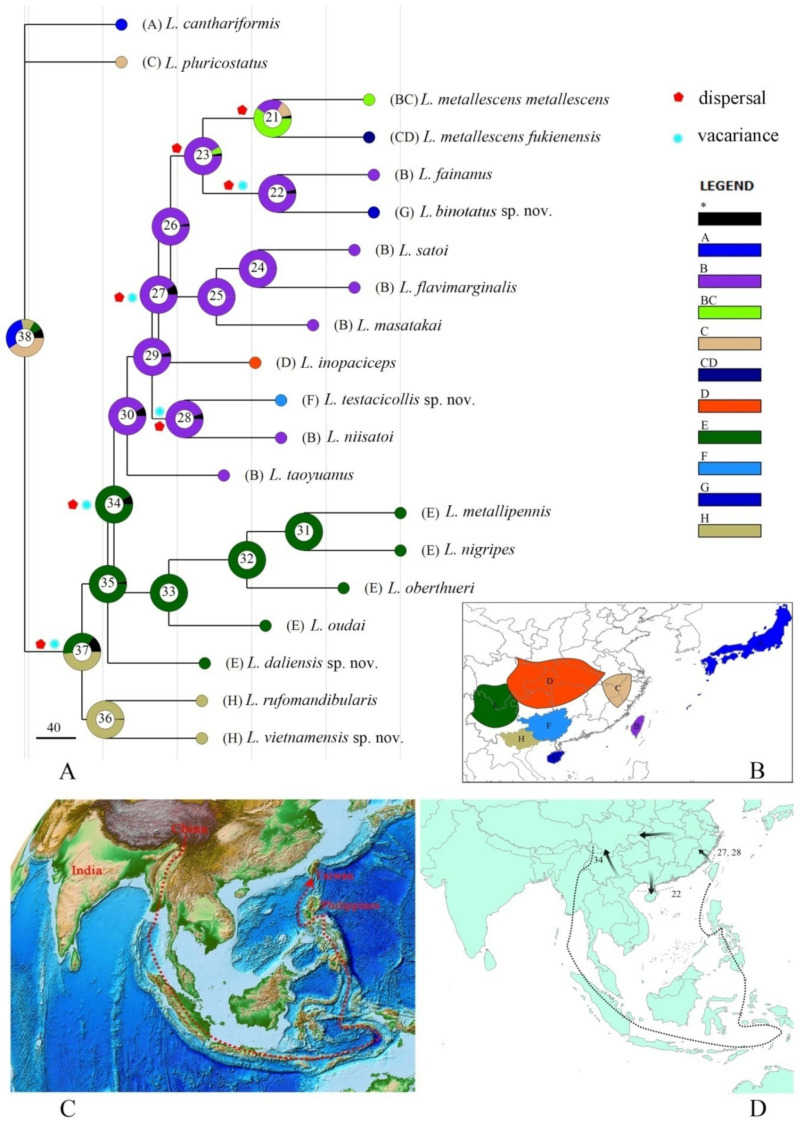
(**A**) Reconstruction of ancestral state in spatial distribution of *Lycocerus* *fainanus* species group; (**B**) Biogeographical areas: A: Japan; B: Taiwan; C: southeast China; D: central China; E: southwest China; F: Guangxi; G: Hainan; H: northern Vietnam. (**C**) Drift trajectory of Taiwan (revised from NOAA; derived from Liang, 2013); (**D**) Dispersal routes (indicated by arrows) and vicariance (by dotted lines) events in the biogeographical history of *L. fainanus* species group.

According to the distribution pattern of the species, eight regions were identified and roughly corresponding to the biogeographical regions and continents: A: Japan; B: Taiwan; C: southeast China (including Fujian, Zhejiang, Anhui); D: central China (Hunan, Hubei, Shaanxi, Guizhou, Chongqing, and eastern Sichuan); E: southwest China (including western Sichuan and northern Yunnan); F: Guangxi; G: Hainan; H: northern Vietnam (Figure 7B).

Combined with the phylogenetic analyses (Supplementary Figures S7 and S8) and the biogeographical regions, the dispersal and radiation processes in the *L. fainanus* species group were explored based on the BBM analysis. The results are shown in Figure 7A. There were seven dispersals (nodes 37, 34, 27, 28, 22, 23, and 21), five vicariance (nodes 37, 34, 28, 27, and 22), and the centers of origin were north Vietnam and southwest China. Subsequently, two dispersal routes occurred (Figure 7D): the first originates from the continent to Taiwan, and the second started from Taiwan, where the species dispersed to the continent. The divergence between *L. testacicollis* sp. nov. and *L. niistatoi*(node 28), also *L. binotatus* sp. nov. and *L. fainanus* (node 22) were caused by dispersal and vicariance. That between *L. metallescens* *fukienensis* and *L. metallescens* *metallescens* (node 21) was caused only by dispersal.

## 4. Discussion

### 4.1. Definition of L. Fainanus Species Group

The number of the species in the *L. fainanus* species group was increased from 9 to 18. With more species added to this group, the body size ranges become more extensive, from middle to large-sized (10.0–16.0 mm).

Within this group, the characteristics of tarsal claws could be variable; either all are simple, or each of the pro- and meso-outer claws is equipped with a digitiform tooth in females. Besides, the antennal grooves are often present in males but absent in *L. vietnamensis* sp. nov. and *L. rufomandibularis*, both from North Vietnam. Additionally, except the process is always present in the median lobe, the shapes of aedeagi are quite variable among the species. However, it is certain that these characters are useful for analyzing relationships of the species.

In contrast, the characteristics of the female reproductive system were quite stable in the *L. fainanus* species group, whose diverticulum and spermatheca was relatively long, but the spermathecal duct is short, and spermatheca is provided with a spiral tube. This suggests the female reproductive system is a more reliable character in defining the species group of *Lycocerus* [2], and it is also highlighted in the classification of cantharid subfamilies [22].

No matter how, the status of the species in *Lycocerus* can be clarified if all characters were combined, including the appearance and genitalia of both sexes.

### 4.2. Origin, Specification, and Migration of the L. Fainanus Species Group

The spatial origin is most probably located in northern Vietnam and southwest China, and the divergence between the species of southwest China and Taiwan was caused by dispersal and vicariance. The formation of Taiwan is a result of the India Plate northward extrusion from the China Qinghai–Tibet Plateau [23]. It separated from the latter about 24 Ma ago [24], around the period between the late Oligocene and early Miocene, so the *L. fainanus* species group occurred as early as or in that period. Since its separation, Taiwan traveled around southeast Asia and stayed in the Philippines for a short time, and now it is moving back to China (Figure 7C; Liang, 2013 [23]).

Except for those species of southwest China, the other species from the Chinese mainland all originate from Taiwan. The divergence of *L. metallescens* *metallescens* (southeast China and Taiwan) and *L. metallescens* *fukienensis* (southeast and central China) was caused only by dispersal; this suggests the connection of Chinese mainland and Taiwan indeed happened in history, as noted by others. Land bridges connecting the Chinese mainland and Taiwan have been proposed five different times, and the latest connection occurred 20,000 years ago during the late Pleistocene [25,26,27]. Therefore, there were chances for the species from the Chinese mainland and Taiwan to communicate with each other, which show close relationships. The divergence between *L. niisatoi* (Taiwan) and *L. testacicollis* sp. nov. (Guangxi) and *L. fainanus* (Taiwan) and *L. binotatus* sp. nov. (Hainan) were probably caused by the vicariance of the formation of the Taiwan Strait, respectively. It is also possible that the latter was caused by the formation of the Qiongzhou Strait.

Hainan was connected to Vietnam and Guangxi, China in the Eocene, then moved and rotated to the southeast and finally reached its present position [28]. After it broke away from the continent, it drifted slowly and connected with the mainland several times during the glacial period due to the decline of sea level [29]. Thus, it was possible for the species to migrate from Taiwan to the continent and then to Hainan.

Currently, there is only one fossil record of *Lycocerus*, *L. guttula* (J. Zhang 1989 [30]) originally in *Cantharis* L., 1758 from Middle Miocene (16.0–11.6 Ma), Shanwang Formation, China, and was attributed to the *L. oedemeroides* species group by Fanti [31]. Unfortunately, it is difficult to estimate the timing of origin for the *L. fainanus* species group because of the lack of direct fossil evidence and molecular data.

## Supplementary Materials

The following are available online at https://www.mdpi.com/article/10.3390/insects12050445/s1, Figure S1: Aedeagus (A, D. ventral view; B, E. dorsal view; C, F. lateral view): A–C. Lycocerus fainanus (Pic, 1910); D–F. L. taoyuanus (Wittmer, 1983). Scale bars: 1.0 mm; Figure S2: Aedeagus (A, D. ventral view; B, E. dorsal view; C, F. lateral view): A–C. Lycocerus inopaciceps (Pic, 1926); D–F. L. metallescens fukienensis (Wittmer, 1954). Scale bars: 1.0 mm; Figure S3: Aedeagus (A, D. ventral view; B, E. dorsal view; C, F. lateral view): A–C. Lycocerus oberthueri (Gorham, 1889); D–F. L. oudai (Švihla, 2004). Scale bars: 1.0 mm; Figure S4: Aedeagus (A, D. ventral view; B, E. dorsal view; C, F. lateral view): A–C. Lycocerus metallicipennis (Fairmaire, 1887); D–F. L. nigripes (Wittmer, 1995). Scale bars: 1.0 mm; Figure S5: Abdominal sternite VIII of female, ventral view: A. Lycocerus fainanus (Pic, 1910); B. L. taoyuanus (Wittmer, 1983); C. L. inopaciceps (Pic, 1926); D. L. metallescens fukienensis (Wittmer, 1954); E. L. oberthueri (Gorham, 1889); F. L. oudai (Švihla, 2004); G. L. metallicipennis (Fairmaire, 1887); H. L. nigripes (Wittmer, 1995). Scale bars: 0.5 mm; Figure S6: Female internal genitalia, lateral view: A. Lycocerus fainanus (Pic, 1910); B. L. taoyuanus (Wittmer, 1983); C. L. oberthueri (Gorham, 1889); D. L. oudai (Švihla, 2004); E. L. nigripes (Wittmer, 1995). Scale bars: 1.0 mm; Figure S7: Phylogenetic tree of the Lycocerus fainanus species-group based on morphological characters by Maximum Parsimony (MP) analysis; Figure S8: Phylogenetic tree of the Lycocerus fainanus species-group based on morphological characters by Neighbor-jointing (NJ) analysis; Table S1: List of characters and states used in phylogenetic analysis; Table S2: The matrix of character states of Lycocerus fainanus species-group in phylogenetic analysis; Table S3: The distribution information for all species of *Lycocerus* *fainanus* species-group.
