# Supplementary material for: Spatial Origin and Diversification of the Lycocerus fainanus Species Group (Coleoptera, Cantharidae), with Descriptions of Four New Species from China and Vietnam
Source: Insects. 2021 May 13;12(5):445. doi: 10.3390/insects12050445 (PMC8153118; doi:10.3390/insects12050445)
Supplement: Supplementary file 1 [file insects-12-00445-s001.zip › insects-1209901-supplementary.pdf]

Article

Spatial origin and diversification of the *Lycocerus fainanus* species-group (Coleoptera, Cantharidae), with descriptions of four new species from China and Vietnam

Huacong Xi[1], Younan Wang[1], Tong Liu[1], Xingke Yang[2], Haoyu Liu[1*] and Yuxia Yang[1*]

[1] The Key Laboratory of Zoological Systematics and Application, School of Life Science, Institute of Life Science and Green Development, Hebei University, Baoding 071002, Hebei Province, China; xihuacong5253@163.com (H. X.); wangyounan@stumail.hbu.edu.cn (Y. W.); liutong@stumail.hbu.edu.cn (T. L.)
[2] Key Laboratory of Zoological Systematics and Evolution, Institute of Zoology, Chinese Academy of Sciences, Beijing 100101, China; yangxk@ioz.ac.cn (X. Y.)
* Correspondence: liuhy@hbu.edu.cn (H. L.); yxyang@hbu.edu.cn (Y. Y.)

Supplementary tables

Table S1 List of characters and states used in phylogenetic analysis

No.	Characters	States
0	Body shape (length vs. width)	(0) stout (less than 4.0 times); (1) slender (more than 4.0 times).
1	Head coloration	(0) uniformly black; (1) bicolored, mixed black and orange; (2) uniformly orange.
2	Vertex coloration	(0) black; (1) orange.
3	Eyes shape	(0) small; (1) large.
4	Terminal maxillary palpi shape	(0) stout and short, expanded apicad; (1) cultellate.
5	Antennae shape	(0) filiform; (1) serrate.
6	Grooves on middle antennomeres in male	(0) absent; (1) present.
7	Pronotum shape	(0) broad quadrate (wider than long); (1) subqudrate (nearly as long as wide or longer than wide).
8	Pronotum coloration	(0) uniformly black; (1) orange, mixed with black; (2) uniformly orange.
9	Elytral costae	(0) weakly indicated; (1) indicated.
10	Elytral luster	(0) matt; (1) metallic.
11	Elytra shape	(0) parallel; (1) dilated posteriorly.
12	Elytra coloration	(0) unicolored; (1) bicolored.
13	Tarsal claws in female	(0) each outer claw of fore and middle legs provided with a digitiform tooth at the base; (1) all simple.

14	Femora coloration	(0) uniformly black; (1) bicolored; (2) uniformly orange.
15	Tibiae coloration	(0) uniformly black; (1) bicolored; (2) uniformly orange.
16	Middle part of posterior margin of female abdominal sternite VIII	(0) weakly emarginated; (1) widely and roundly emarginated; (2) small and triangularly emarginated.
17	Lateroapical angles of female abdominal sternite VIII	(0) rounded; (1) emarginated.
18	Lateral emarginations of female abdominal sternite VIII	(0) simple; (1) membranous.
19	Lateral vs. middle emargination in depth	(0) as deep as; (1) deeper than.
20	Ventral process of parameres	(0) almost straight; (1) bent inwards.
21	Apices of ventral process	(0) simple and rounded; (1) acutely hooked.
22	Dorsal plates of parameres	(0) conjoint; (1) separated.
23	Dorsal plate shape	(0) subparallel; (1) narrowed apicad.
24	Lateral margin of dorsal plate	(0) simple; (1) with one protuberance; (2) with two protuberances.
25	Apical margin of dorsal plate	(0) straight; (1) rounded; (2) protuberant at lateral angles.
26	Inner surface of dorsal plate opposite to apex of laterophyse	(0) nearly simple; (1) arcuately ridged.
27	Dorsal plate vs. ventral process in length	(0) longer than; (1) subequal; (2) shorter than.
28	Emargination between dorsal plate and ventral process	(0) shallow, far from apical margin of basal piece; (1) relatively deep, almost extending to apical margin of basal piece.
29	Laterophyse vs. dorsal plate in length	(0) shorter; (1) subequal.
30	Laterophyse	(0) well developed; (1) reduced (not exceeding over the emargination between dorsal plate and ventral process).
31	Accessory gland of female reproductive organ	(0) expanded at apical portion; (1) not expanded apicad.
32	Diverticulum	(0) short; (1) long.
33	Spermatheca	(0) short; (1) long.

Table S2 The matrix of character states of *Lycocerus fainanus* species-group in phylogenetic analysis

**Species**	**Character states**																																	
	0	1	2	3	4	5	6	7	8	9	10	11	12	13	14	15	16	17	18	19	20	21	22	23	24	25	26	27	28	29	30	31	32	33
L. canthariformis	0	0	0	0	0	0	0	0	1	0	0	0	0	0	0	0	0	0	0	?	0	0	0	0	0	0	0	0	0	0	0	0	0	0
L. pluricostatus	0	0	0	1	1	1	0	1	0	1	0	1	0	0	0	0	1	0	0	1	0	0	1	1	0	1	1	2	0	1	0	1	1	1
L. metallescens metallescens	1	1	0	1	1	0	1	1	1	0	1	0	0	1	2	2	1	0	0	1	1	0	1	0	1	1	1	0	0	0	0	1	1	1
L. metallescens fukienensis	1	1	0	1	1	0	1	1	1	0	1	0	0	1	1	1	1	0	0	1	1	0	1	0	1	1	1	0	0	0	0	1	1	1
L. inopaciceps	1	0	0	1	1	0	1	1	1	0	1	0	0	1	0	0	1	1	0	1	1	0	1	1	1	1	1	0	0	0	0	1	1	1
L. satoi	1	1	1	1	1	0	1	1	1	0	1	0	0	0	1	1	1	0	0	0	1	1	1	1	1	1	1	0	0	0	0	1	1	1
L. masatakai	1	1	0	1	1	0	1	1	1	0	1	0	0	0	2	1	0	0	0	?	1	1	1	1	1	1	1	0	0	0	1	1	1	1
L. flavimarginalis	1	1	0	1	1	0	1	1	1	0	1	0	1	0	1	0	?	?	?	?	1	1	1	1	1	1	1	0	0	0	0	1	1	1
L. testacicollis sp. nov.	1	0	0	1	1	0	1	1	2	0	1	0	0	0	2	0	0	0	0	?	1	0	1	1	1	1	1	1	0	1	0	1	1	1
L. taoyuanus	0	1	0	1	1	0	1	1	1	0	1	0	0	0	0	0	1	0	0	1	1	1	1	1	0	1	0	2	1	0	1	1	1	1
L. fainanus	1	1	0	1	1	0	1	1	1	0	1	0	0	0	?	0	1	0	0	1	1	0	1	0	1	2	1	1	1	1	0	1	1	1
L. bimaculaticollis sp. nov.	1	2	0	1	1	0	1	1	1	0	1	0	0	0	2	2	1	0	0	1	1	0	1	0	1	2	1	1	0	1	0	1	1	1
L. niisatoi	1	0	0	1	1	0	1	1	0	0	1	0	0	1	0	0	1	0	0	0	1	0	1	1	1	1	1	1	0	0	0	1	1	1
L. rufomandibularis	0	0	0	1	1	0	0	1	1	0	1	0	0	0	0	0	1	0	0	0	1	0	1	1	1	1	1	2	0	0	0	1	1	1
L. vietnamensis sp. nov.	0	0	0	1	1	0	0	1	1	0	1	0	0	0	0	0	0	0	0	?	1	0	1	1	1	1	1	0	0	0	0	1	1	1
L. daliensis sp. nov.	0	0	0	1	1	0	1	1	1	0	1	0	0	0	1	0	0	0	0	0	1	0	1	1	1	1	1	0	0	0	0	1	1	1
L. metallipennis	0	0	0	1	1	0	1	1	1	0	1	0	0	0	0	0	2	0	0	1	1	0	1	1	2	1	1	0	0	0	0	1	1	1
L. nigripes	0	0	0	1	1	0	1	1	1	0	1	0	0	0	0	0	2	0	1	1	1	0	1	1	2	1	1	0	0	0	0	1	1	1
L. oudai	0	0	0	1	1	0	1	1	1	0	1	0	0	0	0	0	2	0	0	1	1	0	1	1	1	1	1	0	0	1	0	1	1	1
L. oberthueri	0	0	0	1	1	0	1	1	1	0	1	0	0	0	0	2	2	0	0	1	1	0	1	1	2	1	1	0	0	0	0	1	1	1

Table S3 The distribution information for all species of *Lycocerus fainanus* species-group

Species	Distribution	Longitude	Latitude	Source
***L. bimaculaticollis* sp. nov.**	China: Hainan, Wuzhi Mts, Changjiang.	109.68814	18.90295	This study
	China: Hainan, Jianfengling, Mingfeng Valley.	108.84367	18.74378	—
	China: Hainan, Wuzhi Mts.	109.67	18.90000	—
	China: Hainan, Jianfeng.	108.79245	18.69041	—
	China: Hainan, Jianfengling Nature Reserve, Tianchi.	108.86217	18.74009	—
	China: Hainan, BawanglingNature Reserve, Management Station.	109.08348	19.12119	—
***L. daliensis*sp. nov.**	China: Yunnan, Dali, Cangshan.	100.14351	25.69426	—
	China: Yunnan, Dali, Zhonghefeng.	100.14329	25.68342	—
***L. vietnamensis* sp. nov.**	Vietnam: Sa Pa Distr, Lao Cai, Fan Si Pan.	103.77083	22.34944	—
	Vietnam: Sa Pa Distr, Lào Cai.	103.84409	22.33712	—
***L. testacicollis*sp. nov.**	China: Guangxi, Wuming, Damingshan.	108.27567	23.15488	—
L. taoyuanus	China: Taiwan, Yilan Hsien, Nanshan.	121.77102	25.03619	—
	China: Taiwan, Nantou Hsien, Sungkang.	121.16873	24.07408	—
	China: Taiwan, Taoyuan Hsien, near Chihtuan.	121.23888	24.91073	[S1]
	China: Taiwan, Taoyuan Hsien, near Ssuling.	121.38960	24.83224	—
L. fainanus	China: Taiwan, Nantou Hsien, Shihtyutou.	120.76276	23.61529	This study
	China: Taiwan, Nantou Hsien, Lien Hwa chi.	120.69496	23.50532	[S2]
	China: Taiwan, Nantou Hsien, Nanshanchi.	120.89164	23.92317	[S1]
	China: Taiwan, Nantou Hsien, Fenchihu.	121.06904	24.02365	—
	China: Taiwan, Nantou Hsien, Meifeng.	121.17394	24.08993	—
	China: Taiwan, Nantou Hsien, Tungpu.	120.93081	23.56148	—
	China: Taiwan, Taichung Hsien, Taiko Mts.	120.61355	24.19254	—
	China: Taiwan, Nantou Hsien, Baibara.	121.25552	24.97539	—
	China: Taiwan, Nantou Hsien, Hori.	121.19554	25.06100	—

	China: Taiwan, Nantou Hsien,Wushe.	121.12493	24.02137	—
	China: Taiwan, Nantou Hsien, Yingfeng.	121.23136	24.11636	—
	China: Taiwan, Taoyuan Hsien, Chihtuan.	121.23888	24.91073	—
L. metallicipennis	China: Sichuan, Xiangcheng, Xiangbala.	99.80024	28.93387	This study
	China: Sichuan, Xiangcheng.	99.81129	28.92928	—
	China: Sichuan, Shimian.	102.29139	29.27479	—
	China: Yunnan, Lijiang, Yulongxue Mts.	100.17543	27.09833	—
	China: Yunnan, Zhongdian, Xiaozhongdian.	99.78693	27.60153	—
	China: Yunnan, Weishan.	100.21423	25.13244	[S3]
	China: Yunnan, Yulong Mts.	100.15423	27.14234	—
	China: Yunnan, Lijing.	100.22395	26.87216	—
	China: Yunnan, Jizushan Mts.	100.21324	25.58342	—
	China: Sichuan, Emei Shan.	103.18333	29.58333	[S2]
	China: Yunnan, Lushui.	98.86184	26.05418	—
	China: Yunnan, Lushui, Pianman.	98.61412	26.00853	—
L. inopaciceps	China: Shaanxi, Liubaxian.	106.91933	33.61512	This study
	China: Shaanxi, Liuba, Miaotaizi.	106.84359	33.67906	—
	China: Shaanxi, Fengxian, Heigou.	106.62096	33.93341	—
	China: Shaanxi, Liuba, Zaomulan.	106.85317	33.68856	—
	China: Sichuan, Kwanhsien [now Kangding]	103.64394	30.98796	[S4]
	China: Shaanxi, Lueyang.	106.15641	33.32846	—
L. oberthueri	China: Sichuan, Litang.	100.11644	29.96628	This study
	China: Sichuan, Yajiang, Jianziwan Mts.	100.85499	30.00723	—
	China: Yunnan, Yongshen.	100.75025	26.68081	—
	China: Yunnan, Chuxiong, Midu.	100.44398	25.51315	—
	China: Sichuan, Shuyong, Litang, Kangge.	100.11644	29.97104	—

	China: Sichuan, Sabdé.	102	29.35000	[S3]
	China: Sichuan, Sabdé-Jiulong, Wuouna.	101.51046	29.00424	—
	China: Sichuan, Sabdé.	101.60211	29.14026	—
	China: Yunnan, Pe Yen Tsin.	100.14246	25.72322	[S2]
L. oudai	China: Sichuan, Emei Mts.	103.33464	29.51596	[S5]
	China: Sichuan, Luding.	102.27364	29.87206	[S2]
L. nigripes	China: Yunnan, Lushui.	98.83636	26.07988	This study
	China: Yunnan, Lushui, Pianma.	98.61413	26.00851	—
	China: Sichuan, Liziping near Shimian,Yaan.	102.33671	29.05169	[S3]
L. satoi	China: Taiwan, Ping Tung, Chun Jin.	120.68847	22.38012	This study
	China: Taiwan, Kaohsiung Hsien, Maolin, Tona.	120.73633	22.9317	[8]
	China: Taiwan, Kaohsiung Hsien, Tengchih, Taoyuan	120.76298	22.97479	—
	China: Taiwan, Kaohsiung Hsien, Tien chi	120.91492	23.27765	—
	China: Taiwan, Kaohsiung Hsien, Tengzhi, Yueshan Dist.	120.73865	23.06246	—
	China: Taiwan, Kaohsiung Hsien, Chunyunshan, Logging Road	120.37247	22.72021	—
	China: Taiwan, Chiayi Hsien, Fenchihu	121.06904	24.02365	—
	China: Taiwan, Pingtung Hsien, Kenting	120.78001	21.94820	—
L. metallescens	China: Fujian, Shaowu, Tachulan.	117.47883	27.33783	[S2]
fukienensis	China: Fujian, Kuatun.	117.66667	27.66667	—
	China: Hubei, Yichang, Houhe.	110.59663	30.19643	This study
	China: Hubei, Shennongjia, Yangrizhen.	110.81957	31.73747	—
	China: Hubei, Yienxian, Wanzhaixiang, banchangcun.	109.91972	30.11167	—
	China: Hubei, Yienxian, Shadaogouzhen, Yangsiqiao.	109.95139	30.15222	—
	China: Hubei, Luoyanghe.	113.78105	30.98103	—
	China: Fujian, Jianyang, Huangkengaotou.	117.68279	27.42652	—
	China: Fujian, Chongan, Xingcun, Sangang.	117.92531	27.63442	—

	China: Fujian, Jianyangshi, Huangkengzhen, Aotoucun, Xianfengling.	117.66061	27.70716	—
	China: Fujian,Wuyishan, Xingcunzhen, Tongmucun, Guadun.	117.64141	27.73362	—
	China: Anhui, Jixixian, Qingliangfeng.	118.86679	30.10498	—
	China: Zhejiang, Qingliangfeng, Tianchi.	119.07579	30.25398	—
	China: Zhejiang, Changhuaxian, Jiufucun.	118.93334	30.09968	—
	China: Zhejiang, Linan, Qingliangfeng.	119.04073	30.13212	—
	China: Zhejiang, Qingliangfeng, Shunxiwu.	118.93569	30.03212	—
	China: Zhejiang, Qingliangfeng, Longtangshan.	118.89869	30.12245	—
	China: Chongqing, Jiangjinshi, Simian Mts.	106.34194	28.59847	—
	China: Chongqing, Jiangjin.	106.26296	29.29149	—
	China: Sichuan, Wanxian, Wangerbao.	104.41229	29.17497	—
	China: Guizhou, Daozhenxian, Sanqiaozhen.	107.51348	29.05437	—
	China: Guizhou, Daozhen, Yuheba.	107.42124	29.06728	—
	China: Guizhou, Daozhenxian, Chengjia Mts.	107.52758	26.64879	—
	China: Hunan, Zhangjiajie, Wulingyuan, Laomuyusuidao.	110.47924	29.29113	—
	China: Hunan, Zhangjiajie, Wulingyuan, Wenfengshan, Wangshanzhuang.	110.58983	29.33864	—
L. metallescens	China: Jiangxi, Kiukiang.	116.00464	29.70428	—
metallescens	China: Fujian, Kuatun.	117.66666	27.66666	—
	China: Zhejiang, Hangchow.	120.155401	30.264874	—
	China: Zhejiang, Tienmu Mts.	119.61468	30.42223	—
	China: Taiwan, Taoyuan Hsien, near Chihtuan.	121.23888	24.91073	[S1]
	China: Taiwan, TaoyuanHsien, Shimomura, Ssuling.	121.38960	24.83224	—
	China: Taiwan, Taoyuan Hsien, Shimomura, Kao-i.	121.36781	24.71105	—
L. rufomandibularis	Vietnam: Lao Kay.	103.99956	22.46069	[S3]
	Vietnam: Chapa.	103.96934	22.49176	—
	Vietnam: Sa Pa, Lào Cai.	103.84509	22.33681	[7]

	Vietnam: Deo O Quy Ho.	103.76312	22.36353	—
L. niisatoi	China: Taiwan, Hualien Hsien, Guangfu,lindao,Wanrong.	121.42549	23.66942	—
	China: Taiwan, Taitung Hsien, Matailin Mts.	121.42018	23.41695	—
	China: Taiwan, Yilan Hsien, Chituan, Mingchi,	121.47316	24.65056	—
	China: Taiwan, Hualien Hsien, Taroko.	121.42813	24.20834	—
	China: Taiwan, Pingtung Hsien, Dahan Shan, Chunri Township.	120.75107	22.40809	—
	China: Taiwan, Pingtung Hsien, Dahan Mts Forest Trail.	120.75107	22.40809	—
	China: Taiwan, Yilan Hsien, Tuchang.	121.49643	24.57285	—
L. masatakai	China: Taiwan, Taoyuan Hsien, Mt. Lala Mts.	121.43417	24.73024	[8]
	China: Taiwan, Taipei Hsien,Wulai.	121.55012	24.79156	—
	China: Taiwan, Taoyuan Hsien, Palin-Chihtuan.	121.23384	25.08087	—
	China: Taiwan, Taoyuan Hsien, Sulo.	121.53377	24.6921	—
	China: Taiwan, Taoyuan Hsien, Suling-Chihtuan.	121.38960	24.83224	—
	China: Taiwan, Yilan Hsien, Twolin.	121.75449	24.74888	—
	China: Taiwan, Yilan Hsien, Fushan, botanical.	121.59463	24.75642	—
	China: Taiwan, Yilan Hsien, Fushan,Yuan Mts.	121.72484	24.74418	—
	China: Taiwan, Miaoli Hsien, Chieh-Cheng, Tai-an Hsiang.	120.90887	24.44613	—
	China: Taiwan, Taichung Hsien, Techi.	120.67491	24.16151	—
	China: Taiwan, Taichung Hsien, Wu-ling Farm.	121.30821	24.34543	—
	China: Taiwan, Nantou Hsien, Sungkang-Meifeng.	121.17431	24.08953	—
	China: Taiwan, Nantou Hsien, Sungkang.	121.27954	24.18125	—
	China: Taiwan, Nantou Hsien,Wanta.	121.12706	23.9548	—
	China: Taiwan, Nantou Hsien,Meimu-Holuan.	120.86612	23.83262	—
	China: Taiwan, Chiayi,Hsien,Karapin.	120.81384	23.51445	—
	China: Taiwan, Chiayi Hsien, Fenchihu.	120.69514	23.50533	—
L. flavimargmalis	China: Taiwan, Chiayi Hsien, Fenchihu.	120.69514	23.50533	—

Supplementary figures

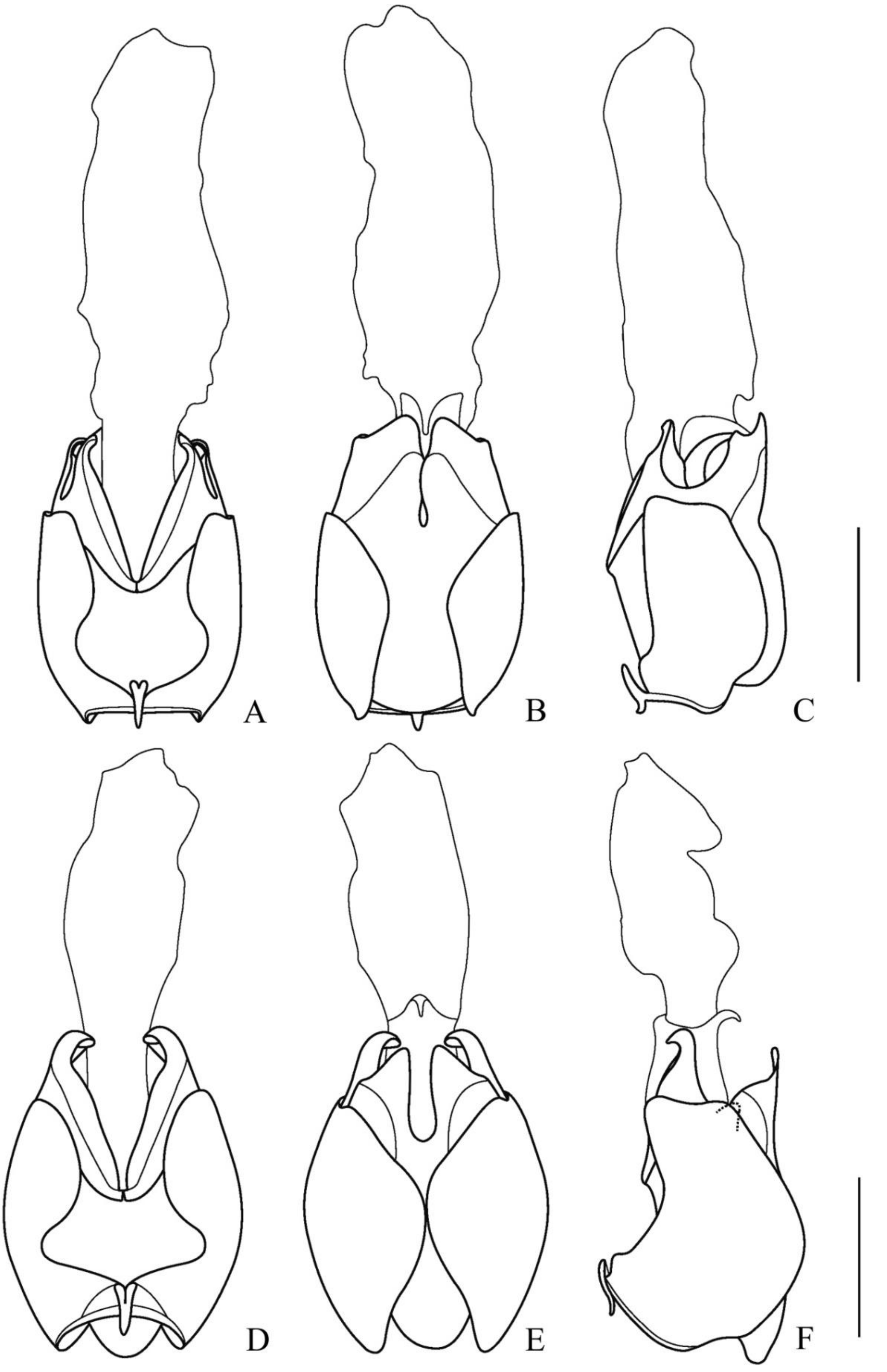

Figure S1 Aedeagus (A, D. ventral view; B, E. dorsal view; C, F. lateral view): A– C. *Lycocerus fainanus* (Pic, 1910); D – F. *L. taoyuanus* (Wittmer, 1983). Scale bars: 1.0 mm.

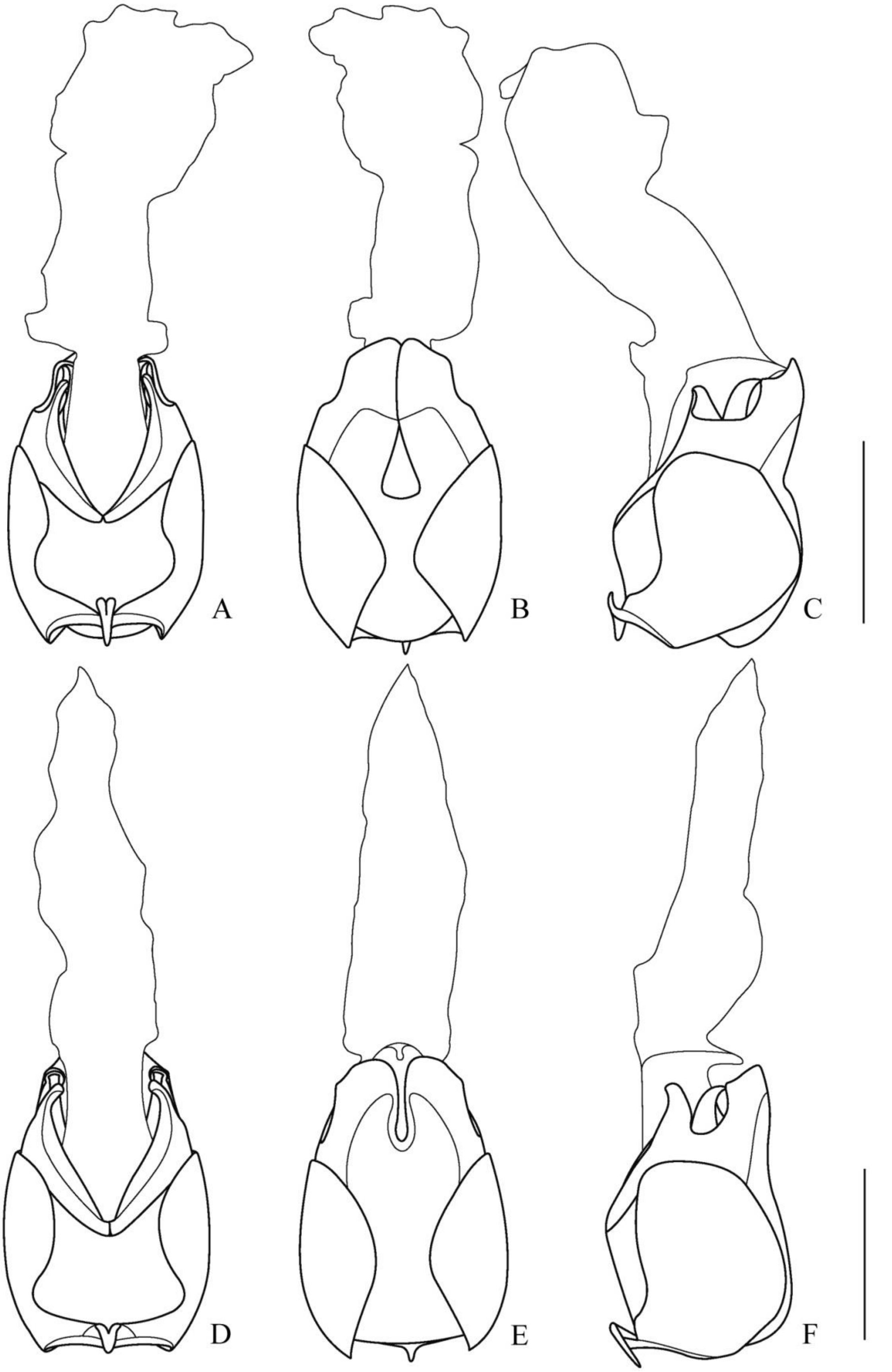

Figure S2 Aedeagus (A, D. ventral view; B, E. dorsal view; C, F. lateral view): A– C. *Lycocerus inopaciceps* (Pic, 1926); D – F. *L. metallescens fukienensis* (Wittmer, 1954). Scale bars: 1.0 mm.

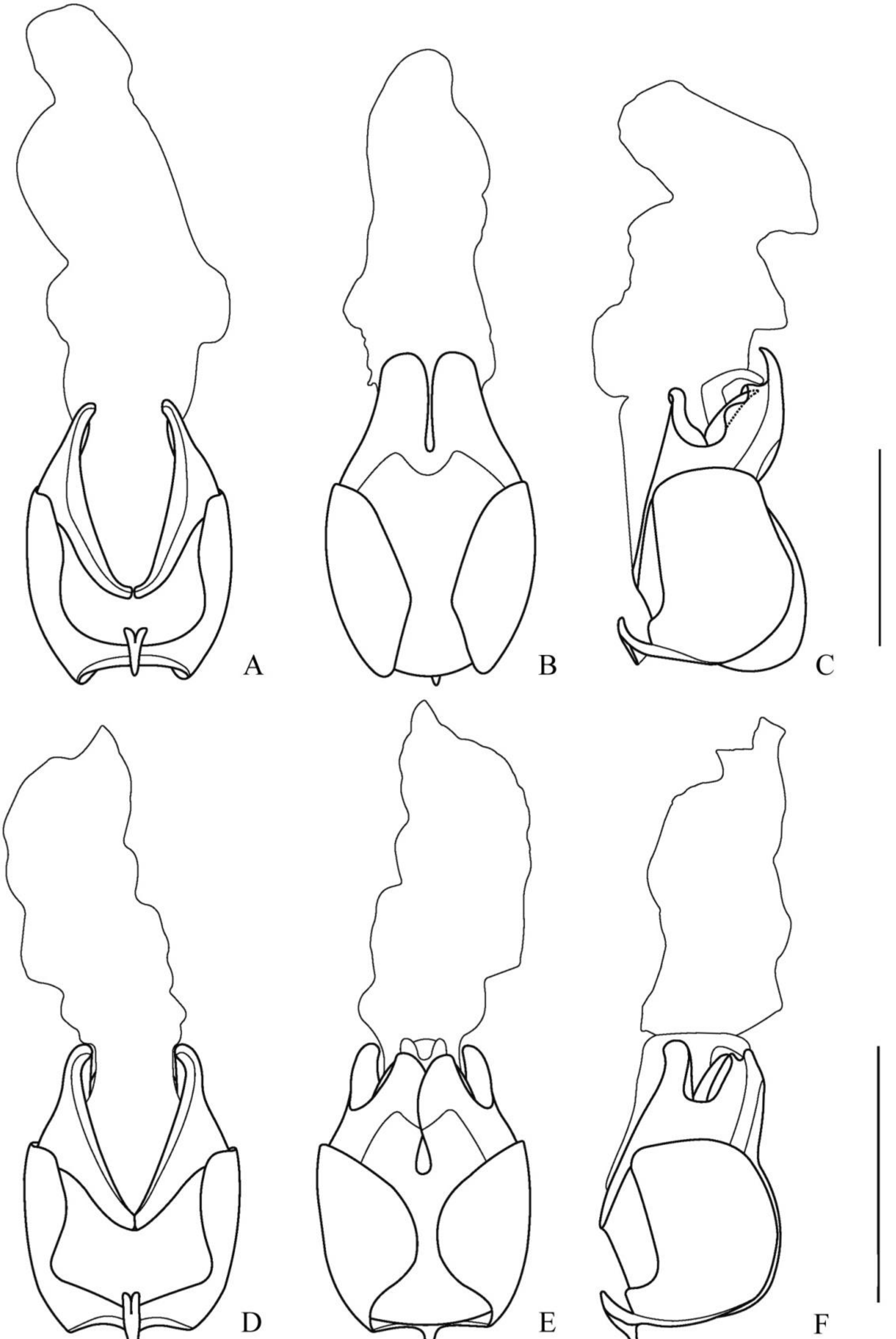

Figure S3 Aedeagus (A, D. ventral view; B, E. dorsal view; C, F. lateral view): A – C. *Lycocerus oberthueri* (Gorham, 1889); D – F. *L. oudai* (Švihla, 2004). Scale bars: 1.0 mm.

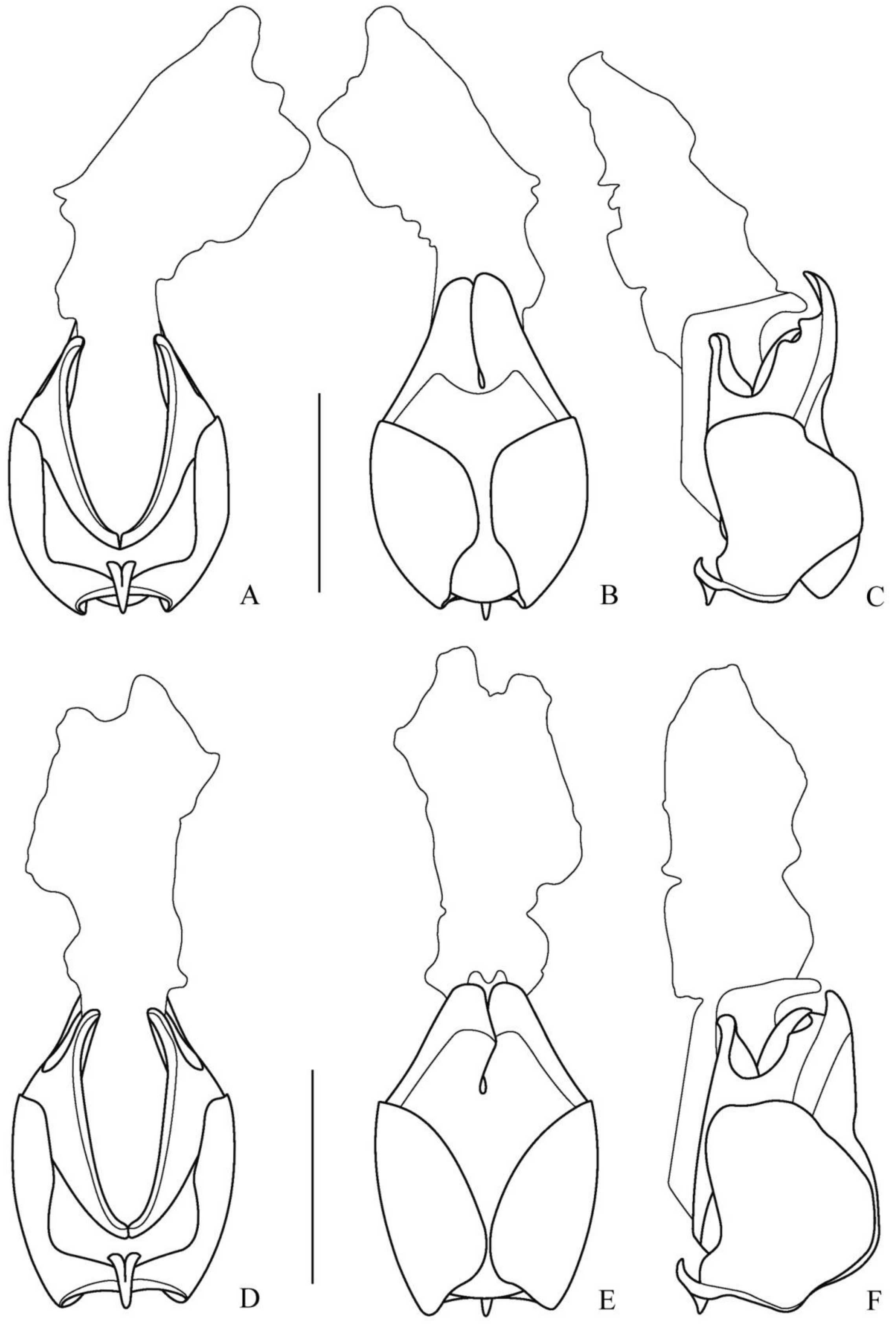

Figure S4 Aedeagus (A, D. ventral view; B, E. dorsal view; C, F. lateral view): A – C. *Lycocerus metallicipennis* (Fairmaire, 1887); D – F. *L. nigripes* (Wittmer, 1995). Scale bars: 1.0 mm.

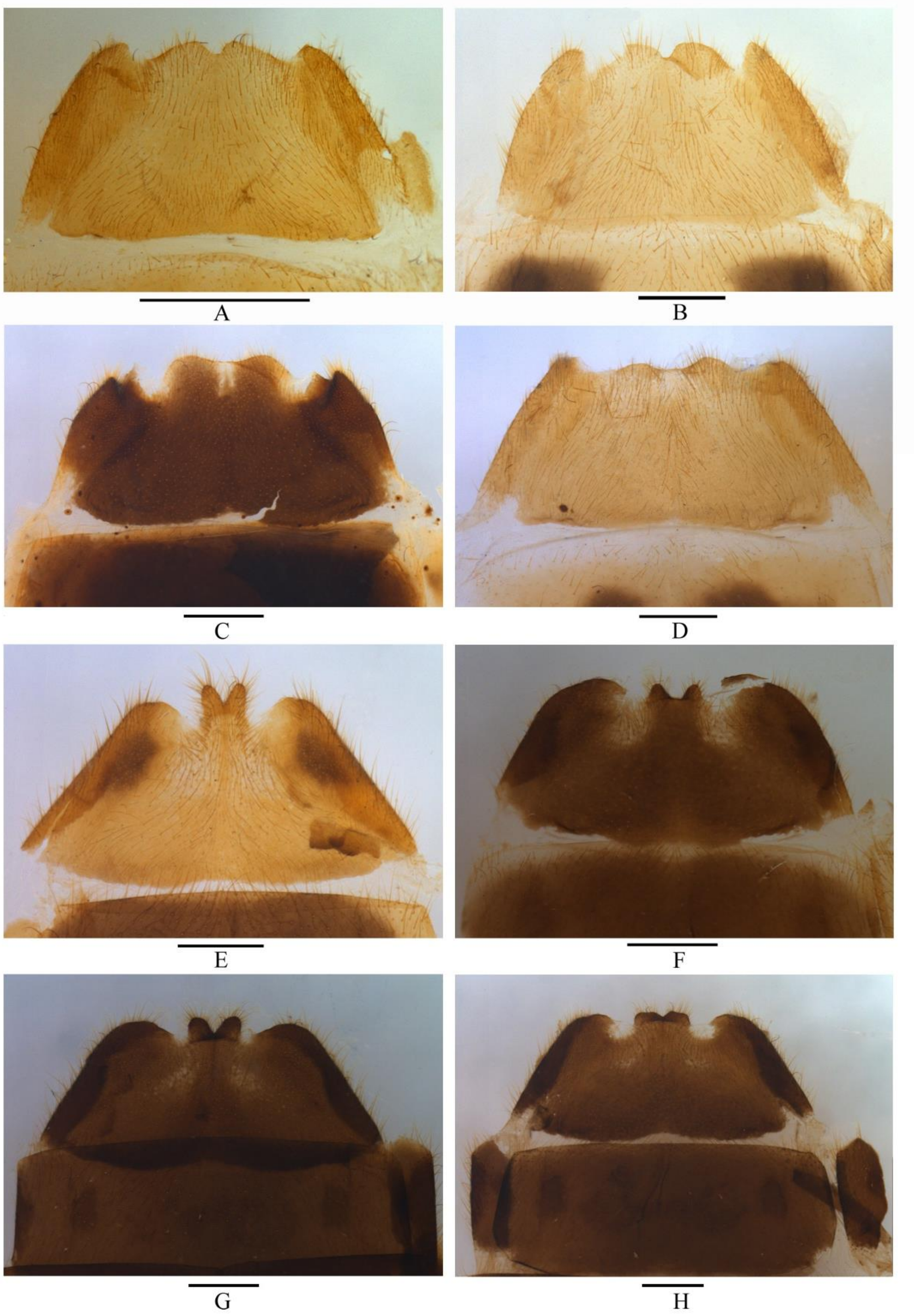

Figure S5 Abdominal sternite VIII of female, ventral view: A. *Lycocerus fainanus* (Pic, 1910); B. *L. taoyuanus* (Wittmer, 1983); C. *L. inopaciceps* (Pic, 1926); D. *L. metallescens fukienensis* (Wittmer, 1954); E. *L. oberthueri* (Gorham, 1889); F. *L. oudai* (Švihla, 2004); G. *L. metallicipennis* (Fairmaire, 1887); H. *L. nigripes* (Wittmer, 1995). Scale bars: 0.5 mm.

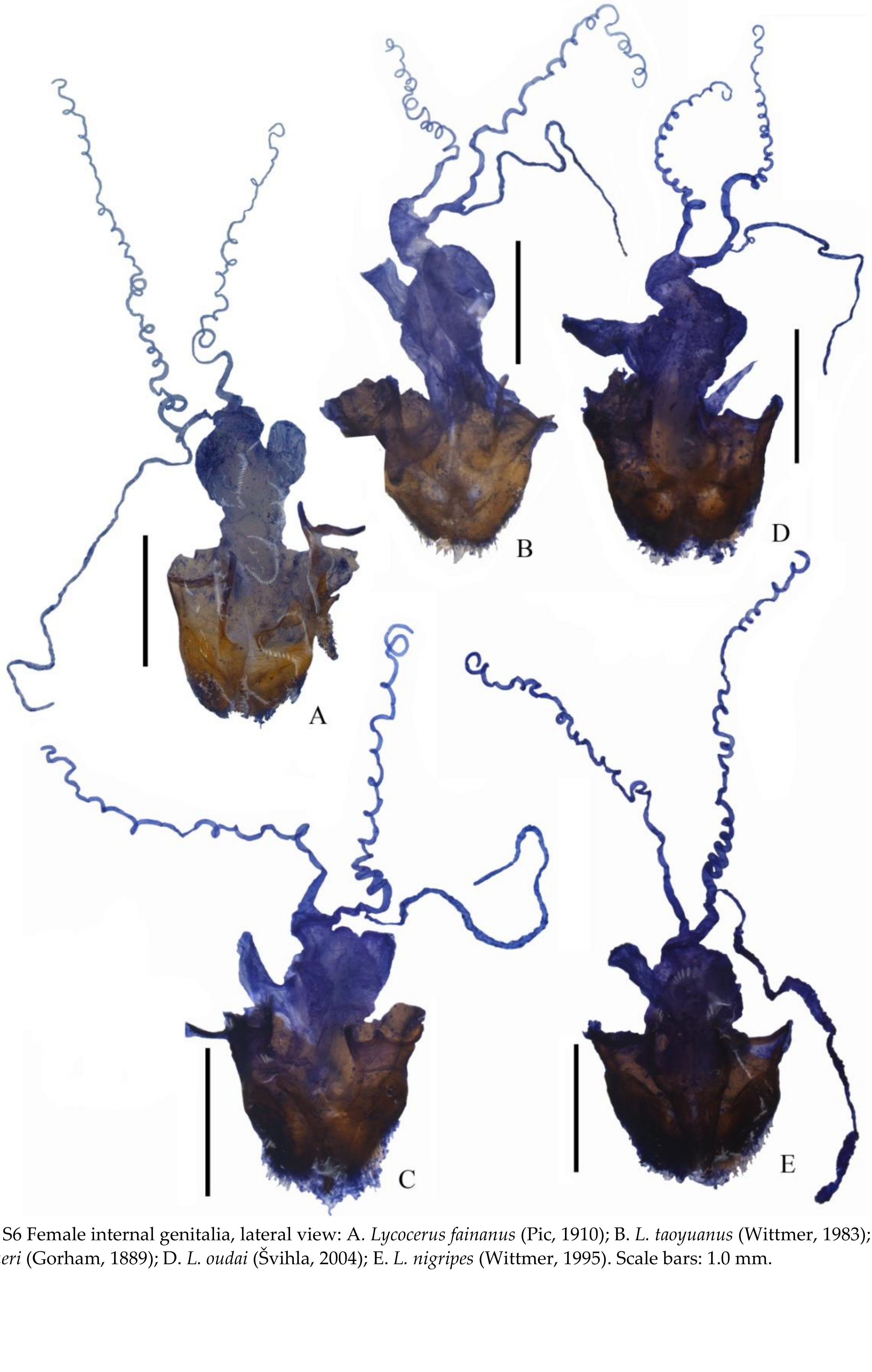

Figure S6 Female internal genitalia, lateral view: A. *Lycocerus fainanus* (Pic, 1910); B. *L. taoyuanus* (Wittmer, 1983); C. *L. oberthueri* (Gorham, 1889); D. *L. oudai* (Švihla, 2004); E. *L. nigripes* (Wittmer, 1995). Scale bars: 1.0 mm.

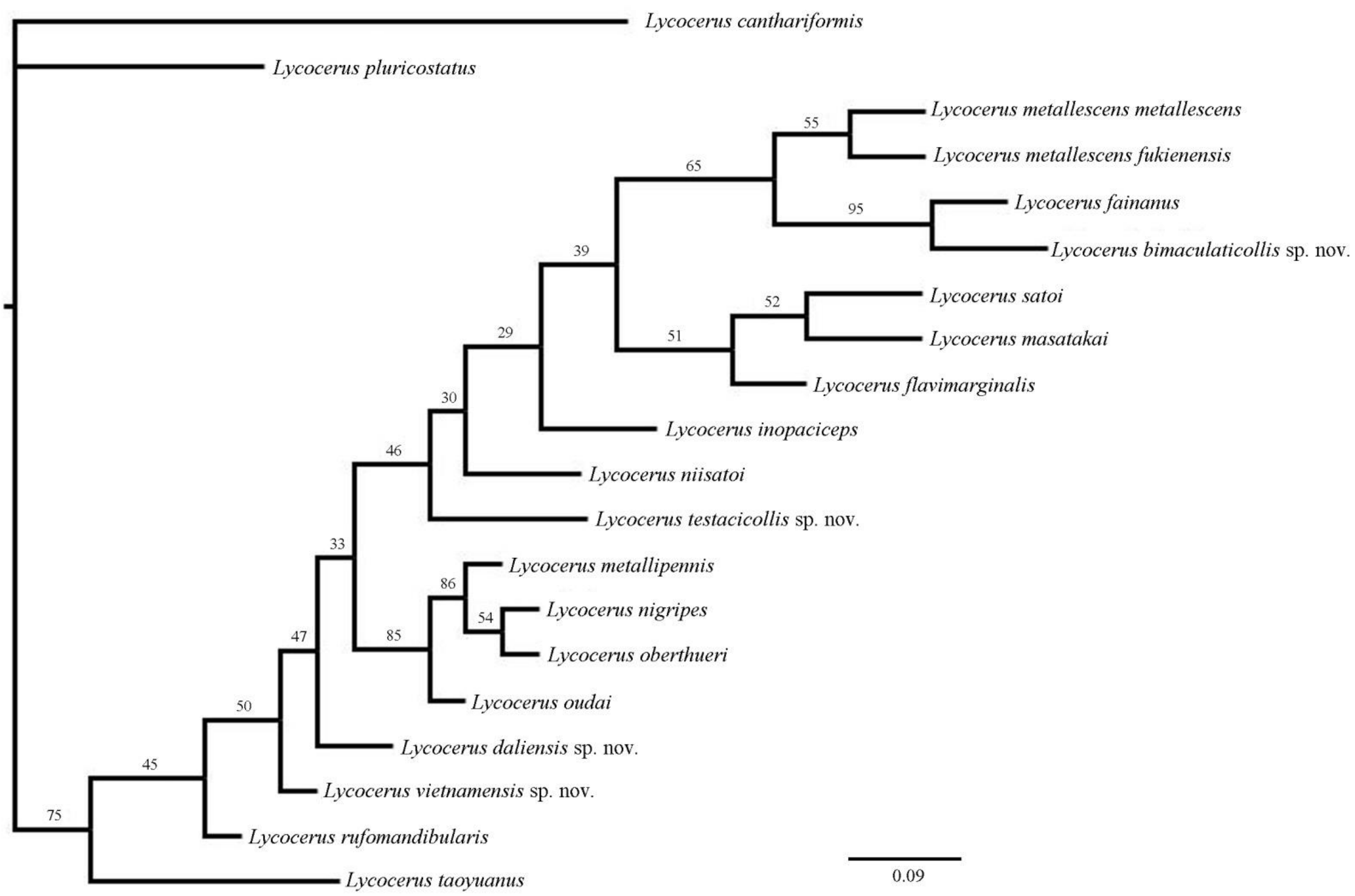

Figure S7 Phylogenetic tree of the *Lycocerus fainanus* species-group based on morphological characters by Maximum Parsimony (MP) analysis

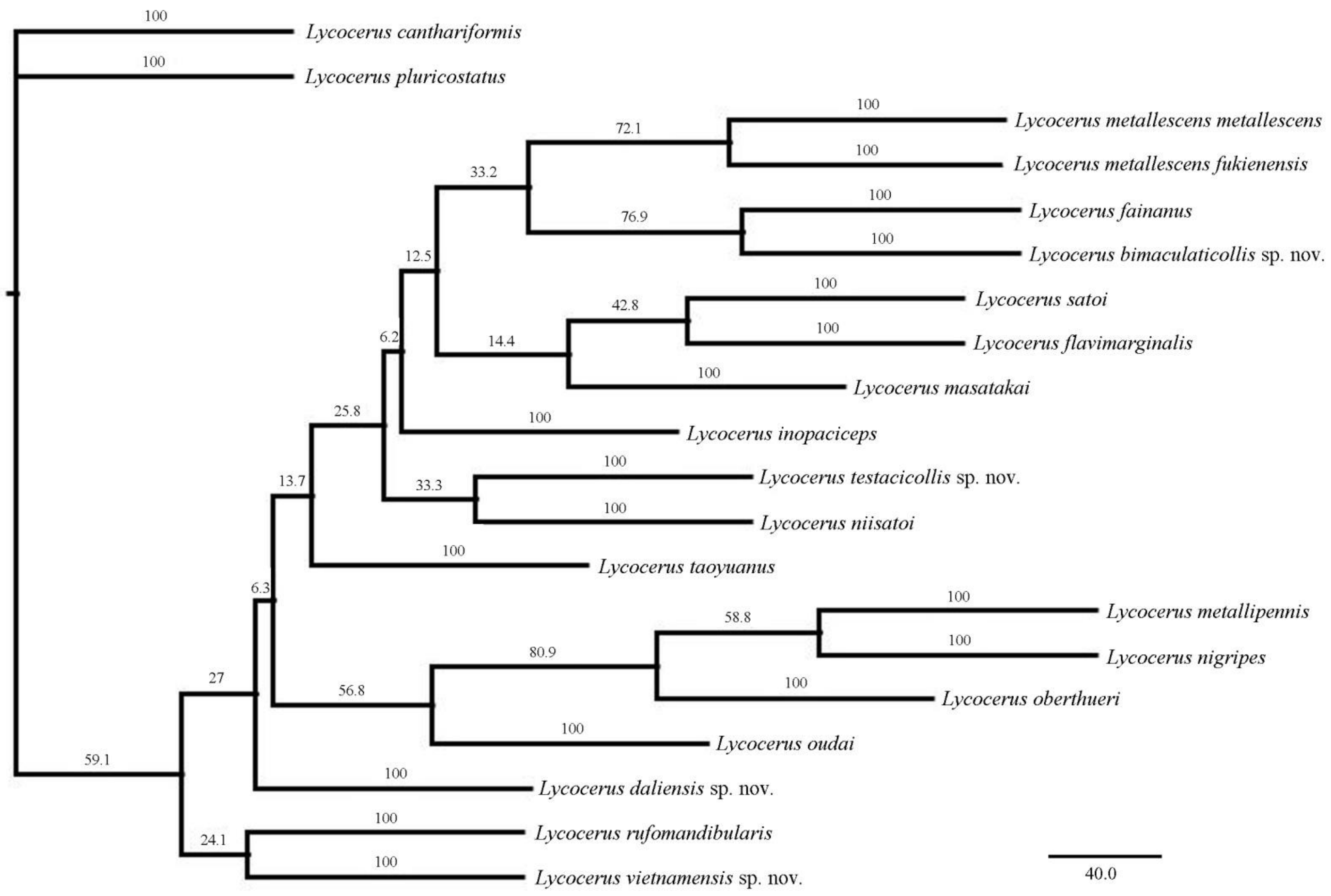

Figure S8 Phylogenetic tree of the *Lycocerus fainanus* species-group based on morphological characters by Neighbor-jointing (NJ) analysis
